# Soybean Leaf Proteomic Profile Influenced by Rhizobacteria Under Optimal and Salt Stress Conditions

**DOI:** 10.3389/fpls.2022.809906

**Published:** 2022-03-24

**Authors:** Gayathri Ilangumaran, Sowmyalakshmi Subramanian, Donald L. Smith

**Affiliations:** Department of Plant Science, McGill University, Montréal, QC, Canada

**Keywords:** salinity, PGPR, leaf proteomics, signaling pathways, stress response, soybean

## Abstract

Soil salinity is a major abiotic stressor inhibiting plant growth and development by affecting a range of physiological processes. Plant growth promoting rhizobacteria (PGPR) are considered a sustainable option for alleviation of stress and enhancement of plant growth, yet their mode of action is complex and largely unexplored. In this study, an untargeted proteomic approach provided insights into growth and stress response mechanisms elicited in soybean plants by *Rhizobium* sp. SL42 and *Hydrogenophaga* sp. SL48 and co-inoculated with *Bradyrhizobium japonicum* 532C. The plants were grown under optimal and salt-stressed conditions up to their mid-vegetative stage; shoot growth variables were increased in the bacteria-treated plants. Shotgun proteomics of soybean leaf tissue revealed that a number of proteins related to plant growth and stress tolerance were modulated in the bacterial inoculation treatments. Several key proteins involved in major metabolic pathways of photosynthesis, respiration, and photorespiration were upregulated. These include photosystem I psaK, Rubisco subunits, glyceraldehyde-3-phosphate dehydrogenase, succinate dehydrogenase, and glycine decarboxylase. Similarly, stress response proteins such as catalase and glutathione S-transferase (antioxidants), proline-rich precursor protein (osmolyte), and NADP-dependent malic enzyme (linked to ABA signaling) were increased under salt stress. The functions of proteins related to plant growth and stress adaptation led to an expanded understanding of plant-microbe interactions. These findings suggest that the PGPR strains regulated proteome expression in soybean leaves through multiple signaling pathways, thereby inducing salinity tolerance, and improving plant growth in the presence of this abiotic stress challenge. Data are available *via* ProteomeXchange with identifier PXD025596.

## Introduction

Salinity is one of the major abiotic stressors, causing detrimental effects on plant growth and development. Soil salinity declines crop productivity and eventually leads to the deterioration of cultivable land and desertification (Abrol et al., 1988; Zorb et al., 2019). Plant growth is affected when the salt concentration in its root zone is above the stress-induction threshold, and it is caused by an initial osmotic phase (water imbalance) and a later ionic phase (ion toxicity). Although roots are the first point of contact in salinity stress, the onset of stress triggers root-to-shoot communication. The responses include stomatal closure, photosynthesis inhibition, oxidative damage, and toxic ion accumulation in the tissues. As a result, leaf area and shoot growth are reduced, and leaf chlorosis and premature senescence are accelerated (Munns and Tester, 2008). Plant salinity tolerance is regulated by a plethora of mechanisms at the molecular, cellular, and physiological levels, throughout the plant’s developmental stages and is reflected in growth rate. These mechanisms have evolved diversely in the plant kingdom so that the degree of salinity tolerance in plants varies among species and genotypes (Chinnusamy et al., 2006).

Soybean [*Glycine max* (L.) Merrill] is an important legume-oilseed crop due to its high protein and oil contents. It is a major source of edible oil, protein, and livestock feed and is cultivated globally. In 2019–20, Brazil (124 million tonnes) and the United States (96.8 million tonnes) were the leading producers, and Canada was the 7th largest producer (6 million tonnes) (SoyStats, 2020). The plant establishes a symbiotic association with *Bradyrhizobium* that dwell in the root nodules and fix atmospheric nitrogen. Soybean enriches soil nitrogen content in agricultural production systems and thus, is included in crop rotations with other arable crops (Zhang and Li, 2003). Expanding soybean cultivation and increasing soybean yield, particularly under stress, has been the major focus of soybean research over the years. Soybean is a glycophyte and is moderately tolerant to salinity stress; seed germination is delayed when exposed to salt and growth traits including seedling emergence, plant height, leaf area, shoot dry weight, nodulation, number of pods, weight per 100 seeds, and seed quality are affected by salinity stress (Phang et al., 2008).

Plant-microbe interactions have crucial functions in plant growth and ecosystem function. Beneficial plant growth promoting rhizobacteria (PGPR) are widely studied and have been shown to elicit tolerance mechanisms that mitigate abiotic stress effects. Inoculation with PGPR modulates plant signaling events involving phytohormones, stress-responses, photosynthesis rate, chlorophyll content, osmolyte accumulation, antioxidant activity, root system architecture, and shoot growth and developmental regulation (Gray and Smith, 2005; Kang et al., 2014). Several studies have reported the influence of PGPR on growth promotion and stress alleviation in soybean with respect to these mechanisms. Soybean seedlings exposed to *Pseudomonas simiae* AU showed significant upregulation of the vegetative storage protein (VSP), gamma-glutamyl hydrolase (GGH), and RuBisCO large chain proteins under salt stress (100 mM NaCl). The plants also had higher proline and chlorophyll contents (Vaishnav et al., 2015). Inoculation with *Bacillus firmus* SW5 resulted in higher chlorophyll, proline, glycine betaine, phenolic and flavonoid contents, and antioxidant enzyme activities in soybean plants under salt stress levels of 40 and 80 mM NaCl. Expression of antioxidant enzyme genes, *APX*, *CAT*, *POD*, and Fe-*SOD* (ascorbate peroxidase, catalase, peroxidase, superoxide dismutase) and salt-response genes, GmVSP, *GmPHD2* (plant-homeo-domain gene of DNA binding ability), *GmbZIP62* (transcription factor involved in ABA and stress signaling), *GmWRKY54* (salt and drought stress tolerance), GmOLPb (osmotin-like protein b isoform gene encoding a neutral PR-5 protein), and *CHS* (chalcone synthase involved in the flavonoid biosynthetic pathway) were upregulated in the salt-stressed plants (El-Esawi et al., 2018).

Soybean plants inoculated with *B. thuringiensis* showed greater stomatal conductance and transpiration rates than the control plants under drought stress. Further, the plants, along with those inoculated with *B. subtilis* and *B. cereus*, showed differential expression of the stress-responsive genes *GmDREB1D* (dehydration-responsive element binding), *GmEREB* (ethylene-responsive element binding), *GmP5CS* (Δ^1^-pyrroline-5-carboxylase synthetase), and *GmGOLS* (galactinol synthase) (Martins et al., 2018). Halotolerant PGPR strains inoculated onto soybean resulted in higher antioxidant enzyme activity, K^+^ uptake, chlorophyll content, and plant growth but decreased ABA level under 200 mM NaCl. The expression of *GmST1* (salt-tolerance 1) and *GmLAX3* (auxin resistant 3) were upregulated in the inoculated seedlings (Khan et al., 2019a). One of the PGPR, *Arthrobacter woluwensis* AK1 increased antioxidant activities and decreased Na^+^ uptake in soybean plants grown under 100 and 200 mM NaCl. Further, the inoculated plants showed upregulation of *GmLAX1* (auxin resistant 1), *GmAKT2* (potassium channel), *GmST1* and *GmSALT3* (salt tolerance-associated gene on chromosome 3) and downregulation of *GmNHX1* (Na^+^/H^+^ antiporter) and *GmCLC1* (chloride channel) (Khan et al., 2019b). It is unsurprising that many of these studies used soybean leaf tissue to elucidate the mechanisms of plant salinity tolerance elicited by PGPR as leaves exhibit clear symptoms of stress and stress responses.

*Amphicarpaea bracteata* (hog peanut) is a legume, native to North America and the closest relative of soybean in eastern North America (Marr et al., 1997). In an earlier study, bacteria were isolated from the root nodules of *A. bracteata* and inoculated onto soybean and screened based on their ability to improve plant growth and salinity tolerance (Ilangumaran et al., 2021). Two isolates, *Rhizobium* sp. SL42 and *Hydrogenophaga* sp. SL48, co-inoculated with *Bradyrhizobium japonicum* 532C were shown to increase plant growth and development under optimal and salt-stressed conditions in a greenhouse setting. The bacteria are currently evaluated for their capacity to enhance soybean growth under field conditions, to be potentially applied as inoculants in soybean crop production systems. However, it is imperative to understand the plant mechanisms regulated by the strains and the function of plant-microbe interactions causing enhanced growth and stress tolerance of soybean. In this present study, we used a proteomic approach to analyze growth and stress related responses elicited in the leaf tissue of soybean plants at their mid-vegetative stage, grown in a controlled environment under both optimal and salt-stressed conditions. We hypothesize that the analysis of the soybean leaf proteome would reveal the vast network of signaling pathways related to plant growth and stress tolerance mechanisms modulated by the inoculation of *Rhizobium* sp. SL42 and *Hydrogenophaga* sp. SL48 and *B. japonicum* 532C. This approach would also provide a comprehensive understanding of plant-microbe interactions between soybean, *B. japonicum*, rhizobacterial strains SL42 and SL48, as most of the earlier studies had not included the symbiont component which is integral to soybean growth. This is one of the very few reports wherein the overall responses at the proteomic level elicited in the leaves of soybean plants at the vegetative stage due to salinity stress and the roles of nodule-bacteria isolated from a native legume are reported.

## Materials and Methods

### Bacteria Culture Propagation and Inoculation

The bacteria *Rhizobium* sp. SL42, *Hydrogenophaga* sp. SL48 and *B. japonicum* 532 C were grown in YEM broth for 48 h, incubated at 25°C and 150 rpm. The cultures were harvested by centrifugation at 5,000 × *g* for 10 min, room temperature (Awel™ MF 48-R, NuAire, United States) and the supernatant was discarded. The pellet was resuspended in 10 mM MgSO_4_ and the optical density was adjusted to 0.1 at A_600nm_ (Ultraspec 4300 pro UV/Visible Spectrophotometer, Biochrom). Soybean seeds (Absolute RR) were soaked in the bacterial cell suspension at a rate of 500 μL per seed or in 10 mM MgSO_4_ (control) for 30 min. For co-inoculation treatments, equal volume (1:1) of bacterial inoculums were mixed.

### Soybean Growth Conditions and Sample Collection

Bacterized and control seeds (5 seeds per pot) were placed in 15.25 cm pots filled with vermiculite (Perlite Canada Inc., Laval, QC, Canada) treated with 300 mL water or 150 mM NaCl. The pots were placed in a growth chamber (Conviron^®^, Canada) and maintained at 25 ± 2°C and 50% relative humidity. Seedling emergence was counted on 7th DAP (days after planting) and the plants were thinned to one seedling per pot. The plants were irrigated with 300 mL water twice a week (every 3–4 days) and fertilized with 1/2 strength Hoagland’s solution once a week and sampled at 28th DAP. Above ground plant growth variables including plant height, leaf area, shoot fresh weight, and dry weight were measured. Dried tissue samples were ground for elemental analysis, N and P were measured on a flow injection analyzer (FIA) (Lachat QuickChem 8000, Hach^®^ United States) and K, Ca, and Na were measured after dilutions and appropriate modifier addition on an atomic absorption spectrophotometer (AAS) (Varian 220FS). The experiment was repeated four times with eight treatments and six replications for each treatment under optimal and salt-stressed conditions. Three replications were allocated for measuring growth variables and three replications were allocated for protein extraction.

### Shotgun Proteomics

For protein extraction, soybean leaves were harvested, flash-frozen in liquid nitrogen and stored at −80°C. Three samples collected were pooled to form a single biological replicate; each treatment comprised three independent biological replications. The protein was extracted using a plant total protein extraction kit (Sigma-Aldrich, St. Louis, MO, United States).

#### Protein Extraction

Briefly, samples were finely ground in liquid nitrogen and ∼100 mg of sample was transferred to a sterile Eppendorf tube. It was incubated with 1 mL of 80% ice-cold methanol-protease inhibitor cocktail for 20 min in −20°C and centrifuged at 12,000 rpm for 10 min at 4°C. The supernatant was discarded, and the procedure was repeated thrice. The sample was then incubated in acetone and washed twice following a similar procedure to remove pigments and other secondary metabolites. The RW4 (Protein extraction Reagent Type 4) solution was added to the pellet, vortexed for 30 s and incubated for 10 min at room temperature (22°C). After centrifugation at room temperature, the supernatant was collected in a new tube. The protein content was quantified using the Lowry method and samples of 20 μg of protein was dissolved in 20 μL of 1 M urea. The samples were subjected to shotgun proteomic analysis at the Institut de Recherches Cliniques de Montréal (IRCM).

#### Proteome Profiling

Total protein was tryptic digested prior to being subjected to LC-MS/MS using a Orbitrap-Velos instrument (Thermo Fisher, Waltham, MA, United States). Tandem mass spectra were extracted; charge state deconvolution and deisotoping were not performed. MS/MS samples were analyzed using Mascot (Matrix Science, London, United Kingdom; Mascot in Proteome Discoverer 2.4.0.305). Mascot was set up to search the Refseq database Glycine_max (86,460 entries), assuming the digestion enzyme trypsin. Mascot was searched with a fragment ion mass tolerance of 0.020 Da and a parent ion tolerance of 10.0 PPM. Carbamidomethyl of cysteine (+57 on C) was specified in Mascot as a fixed modification. Oxidation of methionine (+16 on M) was specified in Mascot as a variable modification.

#### Criteria for Protein Identification

Scaffold (version Scaffold_4.11.1, Proteome Software Inc., Portland, OR, United States) was used to validate MS/MS based peptide and protein identifications. Peptide identifications were accepted if they could be established at greater than 95.0% probability by the Peptide Prophet algorithm (Keller et al., 2002) with Scaffold delta-mass correction. Protein identifications were accepted if they could be established at greater than 99.0% probability and contained at least 2 identified peptides. Protein probabilities were assigned by the Protein Prophet algorithm (Nesvizhskii et al., 2003). Proteins that contained similar peptides and could not be differentiated based on MS/MS analysis alone were grouped to satisfy the principles of parsimony. Proteins sharing significant peptide sequence similarity were grouped into clusters.

### Statistical Analysis

The experiment was established following a completely randomized design. The data were analyzed using the SAS statistical package v.9.4 (SAS Institute Inc., Cary, NC, United States) with Proc Mixed model at a 95% confidence interval and multiple means comparison was by Tukey’s HSD (honest significant difference) at α = 0.05.

Proteomics data were analyzed using Scaffold v.4 (Proteome software, Inc.) for Fisher’s exact test and fold change of identified/known proteins between two sample categories after normalization (embedded) of the quantitative spectral count. The FASTA files generated were analyzed using OmicsBox (BioBam, Bioinformatics Solutions) and the integrated Blast2GO-Pro and InterProScan web services were used for functional annotation of the proteins and to classify the proteins based on functional domains, enzyme codes (EC), biological processes (BP), molecular functions (MF), and cellular components (CC). Scaffold was also used to generate FASTA, and mzldentML files. The mass spectrometry proteomics data have been deposited to the ProteomeXchange Consortium *via* the PRIDE (Perez-Riverol et al., 2019) partner repository with the dataset identifier PXD025596 and 10.6019/PXD025596.

## Results

### Plant Growth and Elemental Analysis

**FIGURE 1:**
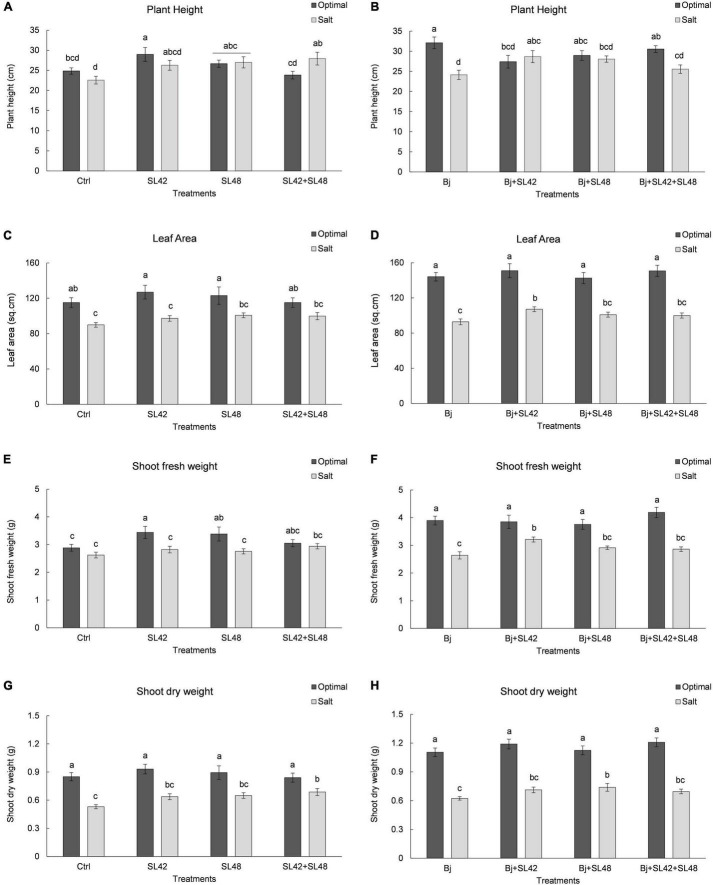
Growth parameters of soybean plants measured at 28th DAP under optimal and salt stress conditions. **(A)** Plant height **(C)** Leaf area **(E)** Shoot fresh weight and **(F)** Shoot dry weight, seeds were treated with 10 mM MgSO_4_ or bacterized with the *Rhizobium* sp. SL42, *Hydrogenophaga* sp. SL48 or co-inoculated (left). **(B)** Plant height **(D)** Leaf area **(F)** Shoot fresh weight and **(H)** Shoot dry weight, seeds were bacterized with *Bradyrhizobium japonicum* (Bj) as control or the strains were co-inoculated with Bj (Right). Values represent mean SE (*n* = 12) and values that share the same letters are not significantly different (a = 0.05).

Seedling emergence of soybean at 7th DAP was lower under salt stress than optimal conditions (Table 1). It was significantly improved by bacterial inoculation treatment with SL42 (*P* = 0.0308) compared to the MgSO_4_ control under optimal conditions. The growth variables of soybean plants were measured at 28th DAP (Supplementary Figure 1). Plant height was significantly increased by treatment with SL42 (*P* = 0.0446) under optimal conditions and for the plants inoculated with SL48 (*P* = 0.0316) and SL42 + SL48 (*P* = 0.0098) under salt stress compared to the control (Figure 1A). Plants inoculated with *B. japonicum* were the tallest under optimal conditions but under salt stress, co-inoculation treatments of *B. japonicum* + SL42 (*P* = 0.0277), *B. japonicum* + SL48 (*P* = 0.0551) resulted in greater plant height than *B. japonicum* alone (Figure 1B). Leaf area was higher than the control for bacterial treatments with SL48 (*P* = 0.0734) and SL42+SL48 (*P* = 0.0569) under salt-stressed conditions, albeit not significant (Figure 1C). Leaf area was significantly higher with the treatment of *B. japonicum* + SL42 (*P* = 0.0464) compared to *B. japonicum* under salinity stress (Figure 1D). Shoot fresh weight was significantly increased by treatment with SL42 (*P* = 0.0293) and SL48 (*P* = 0.0496) under optimal conditions (Figure 1E). Co-inoculation treatment of *B. japonicum* + SL42 had significantly higher (*P* = 0.0227) shoot fresh weight under salt stress than the *B. japonicum* (Figure 1F). Shoot dry weight was significantly increased by treatment with SL42 + SL48 (*P* = 0.0144) than the control under salt-stressed conditions Figure 1G. Co-inoculation treatment of *B. japonicum* + SL42 + SL48 (*P* = 0.0631) resulted in greater shoot dry weight than *B. japonicum* under optimal conditions (Figure 1H). The treatment of *B. japonicum* + SL48 had significantly higher (*P* = 0.039) shoot dry weight compared to the *B. japonicum* under salt stress. Overall, under optimal conditions, SL42 and SL48 bacterial treatments improved plant growth, whereas under salt stress co-inoculation with SL42 + SL48 greatly improved plant growth compared to the control treatment. Growth variables were higher in the *B. japonicum* inoculated treatments than those that had no *B. japonicum*, because of biological nitrogen fixation by *B. japonicum*, which increased shoot N content and boosted vegetative growth. Plant growth was greatly improved by the co-inoculation treatments of *B. japonicum* with SL42 and SL48 compared to *B. japonicum* by itself. Although differences among the co-inoculation treatments were not statistically significant under optimal conditions, they were significant under salinity stress. Elemental analysis revealed the nutrient composition of the soybean shoot tissues (Supplementary Table 1). Nitrogen content was not significantly different between the optimal and salt-stressed plants. Plants under salinity stress had significantly higher (*P* < 0.05) concentrations of phosphorous, potassium and sodium, but lower calcium concentration than the optimally grown plants. The difference among treatments in nutrient elemental concentrations under optimal or salt stress conditions was generally slight. Due to high Na^+^ concentration, the K^+^/Na^+^ ratio was low under salt stress, even though K^+^ concentration was higher.

**TABLE 1:** Seedling emergence rate (%) of soybean at 7th DAP under optimal and salt stress conditions.

Treatments	Optimal	Salt	Treatments	Optimal	Salt
Ctrl	71.7^bc^ ± 5.8	63.3^c^ ± 6.0	Bj	76.7^ab^ ± 6.4	70.0^ab^ ± 3.9
SL42	86.7^a^ ± 2.8	65.0^c^ ± 5.0	Bj + SL42	81.7^a^ ± 4.6	75.0^ab^ ± 3.6
SL48	76.7^abc^ ± 3.3	71.7^bc^ ± 3.9	Bj + SL48	71.7^ab^ ± 5.8	73.3^ab^ ± 6.2
SL42 + SL48	81.7^ab^ ± 4.6	66.7^c^ ± 6.2	Bj + SL42 + SL48	78.3^ab^ ± 4.6	65.0^b^ ± 6.6

*Values represent mean ± SE (n = 12) and values that share the same letters are not significantly different (α = 0.05). Bj, Bradyrhizobium japonicum 532C; SL42, Rhizobium sp. SL42; SL48, Hydrogenophaga sp. SL48.*

### Proteomic Analysis

#### Quantitative Spectra of Soybean Leaf Proteome

**FIGURE 2:**
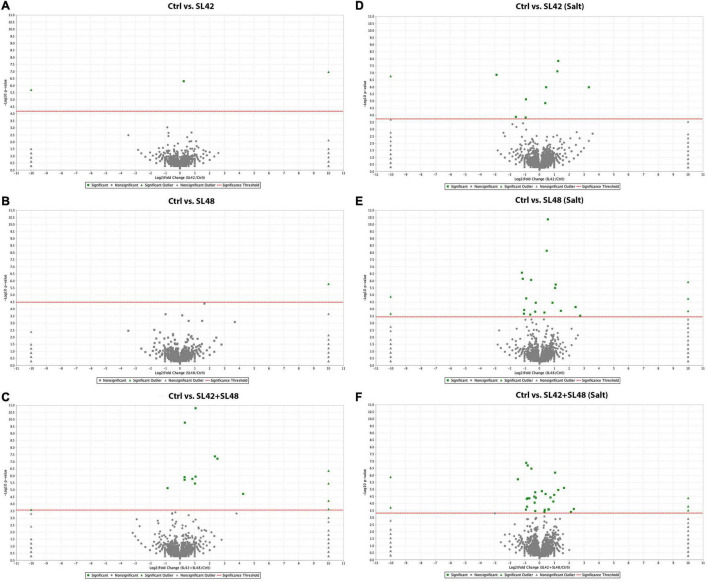
Volcano plots of quantitative spectra comparison between control and bacterial inoculated treatments under optimal and salt-stressed conditions. **(A)** Ctrl vs. SL42 **(B)** Ctrl vs. SL48 **(C)** Ctrl vs. SL42 + SL48 **(D)** Ctrl vs. SL42 (salt) **(E)** Ctrl vs. SL48 (salt) **(F)** Ctrl vs. SL42 + SL48 (salt). Fold change by category, reference control treatment is plotted on X-axis and Fisher’s exact test (Benjamini-Hochberg correction) is plotted on Y-axis.

**FIGURE 3:**
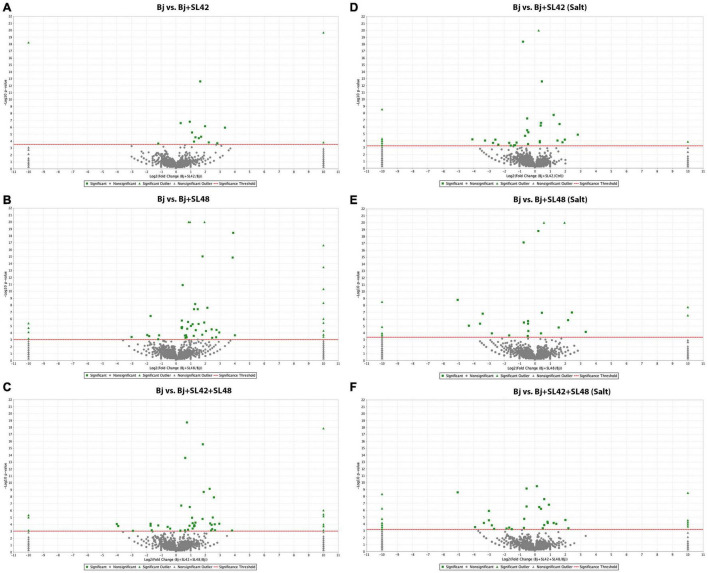
Volcano plots of quantitative spectra comparison between *Bradyrhizobium japonicum* (control) and bacterial co-inoculated treatments under optimal and salt-stressed conditions. **(A)** Bj vs. SL42 **(B)** Bj vs. SL48 **(C)** Bj vs. SL42 + SL48 **(D)** Bj vs. SL42 (salt) **(E)** Bj vs. SL48 (salt) **(F)** Bj vs. SL42 + SL48 (salt). Fold change by category, reference control treatment is plotted on X-axis and Fisher’s exact test (Benjamini-Hochberg correction) is plotted on Y-axis.

To understand the role of the inoculated bacteria on the metabolism and physiology of optimal and salt-stressed soybean plants, a LC-MS/MS based proteome profiling of the total leaf protein extracted was performed. Based on the quantitative value of the spectra, the following number of proteins were identified (Table 2), and the treatment contrasts were analyzed for fold-change after normalization (≥1.2) and Fisher-exact test (*P* ≤ 0.05) to narrow down proteins that were relatively up- or down-regulated. Some of the key proteins might be missed from the analysis due to the very stringent criteria but this allowed for focusing on the proteins that were differentially expressed. Also, for ease of functional interpretation, proteins that were different between the control and the other treatments were analyzed instead of all possible contrasts and volcano plots (significance vs. fold-change) are shown in Figures 2, 3. The number of identified proteins was higher under salt-stressed than under optimum plant growth conditions and they were classified into known, predicted, probable, and uncharacterized proteins.

**TABLE 2:** Total number of proteins identified at 99% protein probability and total spectra at 95% peptide probability, with two minimum peptides.

	Treatments	Protein	Decoy FDP (%)	Spectra	Decoy FDR (%)
Set 1 Optimal	Control	2621	0.00	100193	0.00
	SL42	2524	0.00	99232	0.00
	SL48	2579	0.00	98616	0.00
	SL42 + SL48	2563	0.00	99504	0.00
Set 2 Optimal	Bj	2502	0.00	96259	0.00
	Bj + SL42	2472	0.00	99687	0.00
	Bj + SL48	2530	0.00	103127	0.00
	Bj + SL42 + SL48	2475	0.00	102371	0.00
Set 3 Salt	Control	2672	0.00	96231	0.00
	SL42	2816	0.00	99384	0.00
	SL48	2910	0.00	98642	0.00
	SL42 + SL48	2884	0.00	100621	0.00
Set 4 Salt	Bj	3081	0.00	103148	0.00
	Bj + SL42	2821	0.00	105310	0.00
	Bj + SL48	2808	0.10	104866	0.04
	Bj + SL42 + SL48	2735	0.00	101321	0.01

*Decoy FDP, false discovery proportion; Decoy FDR, false discovery rate; Bj, Bradyrhizobium japonicum 532C; SL42, Rhizobium sp. SL42; SL48, Hydrogenophaga sp. SL48.*

A number of proteins that play an important role in plant growth, development and stress tolerance were significantly upregulated by the bacterial treatments compared to the control (Supplementary Tables 3–6). The commonly upregulated proteins related to cellular function and metabolism included ATP synthases, chlorophyll a-b binding proteins, glyceraldehyde-3-phosphate dehydrogenase A subunit, PSI subunit psaK, RubisCO small and large chains, thioredoxins in the chloroplast, glycine dehydrogenase, NADH dehydrogenases, succinate dehydrogenases in the mitochondria, chaperonins, cytoskeleton proteins (actin and tubulin), peroxisomal enzymes, ribosomal subunits, and proteosome regulatory subunits. The upregulated proteins involved in stress-responses comprised aconitate hydratase, aquaporins, catalases, glutathione S-transferases, heat shock proteins, lipoxygenases, multicystatin, superoxide dismutases, and transketolases. Proteins that were participating in the biosynthesis of alkaloids, carotenes, flavonoids, isoflavonoids, soyasaponins, and other secondary metabolites were also upregulated. Interestingly, specific proteins including PSII protein H, Calvin cycle CP12-2, cucumisin, gibberellin-regulated protein 6 precursor, heme binding 2, and topless-related proteins were upregulated when the strains were co-inoculated with *B. japonicum* relative to the *B. japonicum* control under salt stress.

**FIGURE 4:**
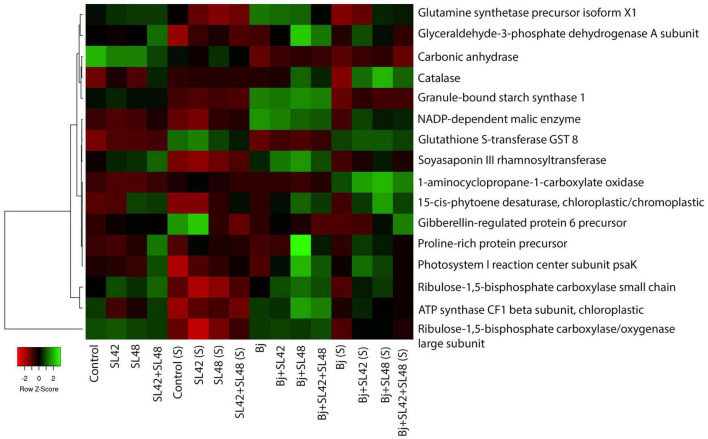
Heat map generated based on quantitative spectral data of selected proteins. Color gradient represents changes of spectral values between treatments and mapped to row scaled data on Y-axis. The small panel in the bottom left corner shows the difference from down-regulated (red) to up-regulated (green). Average linkage clustering and Euclidean distance measurement were applied.

Proteins involved in amino acids, nucleic acids, sugars and starch biosynthesis, nutrient assimilation and mobilization and regulation of plant growth and developmental processes such as ABC transporters, alpha-amylase inhibitor/lipid transfer/seed storage family protein precursor, arginosuccinate lyase, asparagine synthetase, carbamoyl-phosphate synthases, ferredoxins, ferritins, glucose-6-phosphate 1-dehydrogenase, glutamate synthetase, kunitz-type trypsin inhibitor KTI1-like, peroxisomal citrate synthase, polyadenylate-binding proteins, PEP carboxylase, phosphoglycerate kinase, pyruvate kinases, and subtilisin-like proteases were upregulated (Supplementary Tables 7–10). A heatmap generated based on quantitative spectra of major proteins (photosynthesis, antioxidants, and phytohormonal) among treatments under optimal and salt-stressed conditions is given in Figure 4 (Babicki et al., 2016). Moreover, there were unique proteins that were only expressed in the bacterial treatments and not in the controls, such as carboxyl esterase 8, inactive PAP, linoleate 9S-lipoxygenase-2 and 5, lipid transfer protein EARLI 1-like, lysM domain-containing protein, starch synthase enolase, and stress-induced SAM22 (Supplementary Data Sheet 1). The fold change of significantly downregulated proteins was ≤1.0 and so, these were not considered.

#### Functional Classification of Proteins Based on Gene Ontology Categories

**FIGURE 5:**
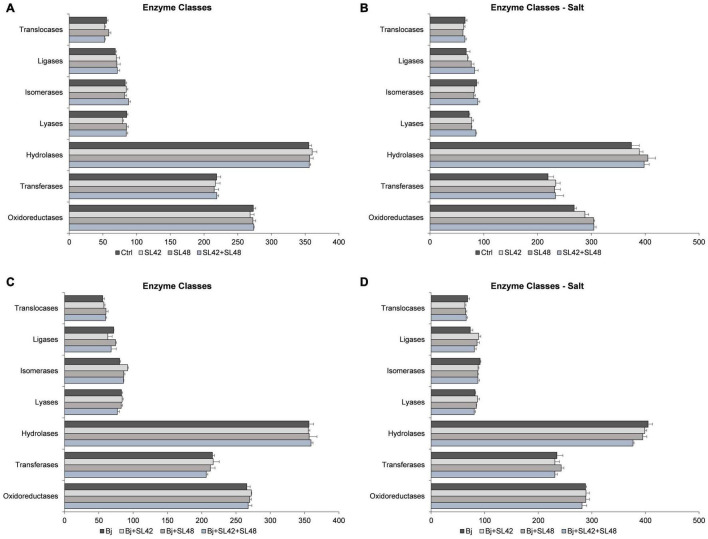
Number of sequences involved in the enzyme classes of the soybean leaf proteome. **(A)** The seeds were treated with 10 mM MgSO_4_ or bacterized with the strains *Rhizobium* sp. SL42, *Hydrogenophaga* sp. SL48 or co-inoculated under optimal **(B)** under salt stress **(C)** seeds were bacterized with *Bradyrhizobium japonicum* (Bj) as control or the strains were co-inoculated with Bj under optimal and **(D)** under salt stress conditions. Values represent mean ± SE (*n* = 3).

Based on Blast2GO pro analysis, the enzymes classes distribution was studied (Figure 5). Some of the enzyme classes were increased under salt stress including oxidoreductases, transferases, hydrolases, and translocases. Under optimal conditions, the difference among the treatments was not more or less than 10 protein sequences. Under salt stress, the oxidoreductases, transferases, and hydrolases were higher in the bacterial treatments than in control. Lyases (17.4%) and ligases (23.3%) were increased, particularly with the treatment of SL42 + SL48. When co-inoculated with *B. japonicum*, little difference was observed among treatments under optimal and salinity conditions. Although, treatment of *B. japonicum* + SL42 increased isomerases (14.5%) under optimal and ligases (21.5%) under salt stress compared to *B. japonicum*.

**FIGURE 6:**
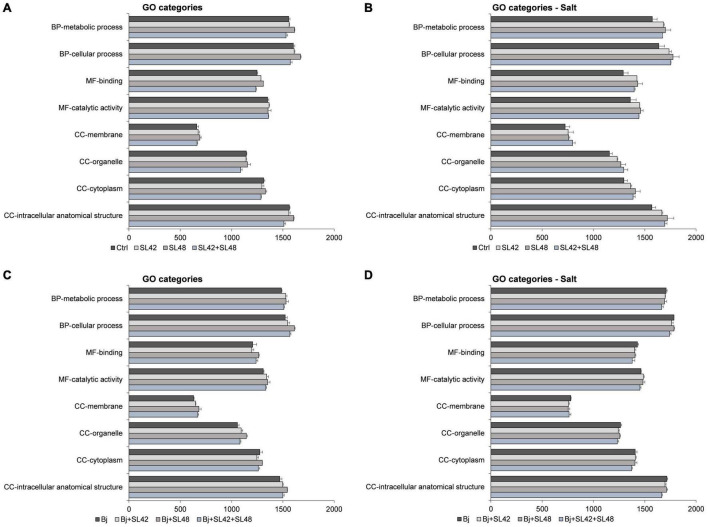
Number of sequences involved in the major GO categories of the soybean leaf proteome. **(A)** The seeds were treated with 10 mM MgSO_4_ or bacterized with the strains *Rhizobium* sp. SL42, *Hydrogenophaga* sp. SL48 or co-inoculated under optimal and **(B)** under salt stress conditions. **(C)** Seeds were bacterized with *Bradyrhizobium japonicum* (Bj) as control or the strains were co-inoculated with Bj under optimal and **(D)** under salt stress conditions. Values represent mean ± SE (*n* = 3).

The Gene Ontology (GO) categories distribution of proteins involved in biological processes, molecular functions, and cellular components were analyzed and the number of proteins associated with almost all functions were increased under salt stress. The major functions (>1000 protein sequences) related to cellular and metabolic processes, binding and catalytic activity, and cellular components including cytoplasm, organelles, membranes, and intracellular structures were all highly upregulated by the bacterial treatments compared to control, but the differences were more prominent under salt stress (>100 sequences) than under optimal conditions. The major GO function proteins were also predominantly upregulated in co-inoculation treatments with *B. japonicum* under optimal conditions (>50 sequences). However, minimal differences (<25 sequences) were observed between the co-inoculation treatments and *B. japonicum* under salt stress (Figure 6).

Similarly, other proteins (<400 sequences) participating in the biological regulation, localization, response to stimulus, detoxification, development, signaling, multicellular organismal processes, interspecies interaction, and reproduction and molecular functions of cellular structures, transport, regulation, translation, and antioxidant activities, were increased by bacterial inoculation compared to the control treatment. The cellular components including cytosol, membrane-protein complex, catalytic complex, ribonucleoprotein, plastid (lumen, stroma, and thylakoid) and mitochondrial proteins were higher in the bacterial treatments than the control (Supplementary Figures 2–4). In the co-inoculation treatments with *B. japonicum*, most functions were upregulated compared to the *B. japonicum* control under optimal conditions. The proteins associated with biological regulation, localization, detoxification, molecular functions, endomembrane system, cell periphery, and extracellular region were down-regulated while signaling, interspecies interaction, and reproduction and cellular components including cytosol, membrane-protein complex, catalytic complex, mitochondrion, respirasome, and supramolecular complex were upregulated in the co-inoculation treatments with *B. japonicum*, relative to *B. japonicum* alone under salt stress. The number of proteins related to other functions were more or less equal and the differences were seldom detectable among treatments. Moreover, when the number of protein sequences involved in a function was less than 20, the differences among treatments were marginal (±3 sequences) (Supplementary Figures 5–8).

## Discussion

The seedling development stage of soybean is more sensitive to salinity than the seed germination stage (Hosseini et al., 2002). This is because the young tissues are affected by osmotic stress due to the high salt concentration in the root zone. In our experiment, seedling emergence was decreased under salt stress and the cotyledons exhibited symptoms such as oxidative browning and wilting on some seedlings. The symptoms of salinity-induced osmotic stress overlap those of drought and cold stress (Zhu, 2002). Shoot growth is limited due to the osmotic imbalance, which affects stomatal conductance, cell expansion in meristems and growth of young leaves (Munns and Tester, 2008). The net photosynthetic rate is reduced and photosynthetic assimilates are utilized for maintenance and survival, rather than biomass accumulation (Munns and Gilliham, 2015). Plant growth was largely reduced by salt stress compared to plants grown under optimal conditions.

Yellowing and senescence of the first two true leaves were observed in salt-stressed soybean plants; this was caused by ionic toxicity and in turn reduced the leaf area (Munns and Gilliham, 2015). The influx of Na^+^ ions affects the concentration of other cations in plant tissues. Salt-tolerant lines of soybean had increased capacity to sustain adequate levels of other nutrient elements required to conduct metabolic functions (Ning et al., 2018). The concentration of N, P, and K were higher under salinity implying that the plants assimilated major nutrients to cope with the negative impacts of salt stress. The plants might have assimilated more K^+^ to maintain ionic homeostasis because of the high Na^+^ content and low Ca_2_^+^ content (cytosolic flux). In salinity affected plants, lower K^+^/Na^+^ ratio suggest increased accumulation of sodium. The tolerance mechanisms include Na^+^ exclusion from the leaf tissues in addition to Na^+^ compartmentalization in vacuoles. Accumulation of compatible solutes (osmolytes) and scavenging of reactive oxygen species (ROS) contribute to enhanced salinity tolerance (Flowers and Colmer, 2015).

**FIGURE 7:**
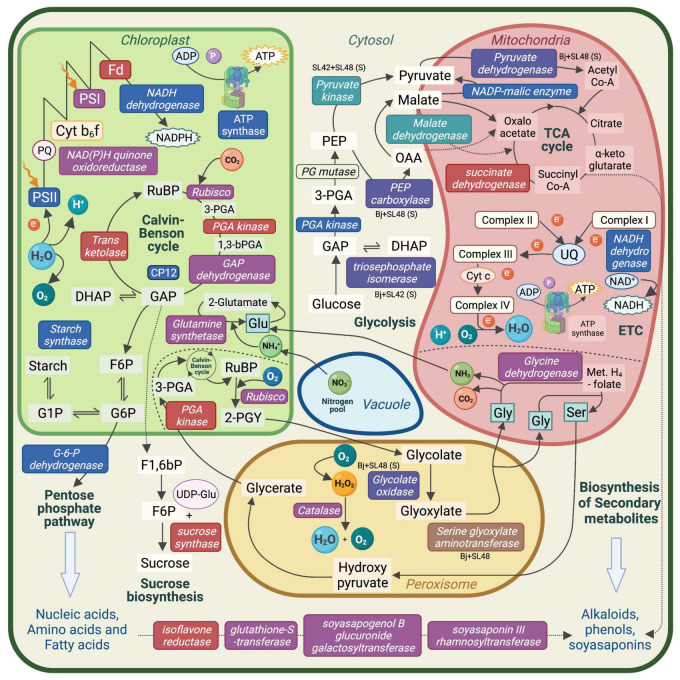
Schematic representation of the major metabolic pathways in a plant cell. Enzymes and molecules involved in photosynthesis, respiration, photorespiration, nutrient assimilation, and biosynthesis pathways that were upregulated in soybean leaf tissue. Upregulated enzymes and molecules by the bacterial co-inoculation treatment, *Bradyrhizobium japonicum* (Bj) + SL42 + SL48 under optimal conditions are indicated in red text box, salt stress conditions are indicated in blue text box, and both conditions are indicated in purple text box. Other specific treatment conditions are indicated as well. PS, Photosystem; Cyt b_6_f, Cytochrome; Fd, Ferredoxin; PQ, Plastoquinone; UQ, Ubiquinone; RuBP, Ribulose bisphosphate; PGA, phosphoglycerate; GAP, Glyceraldehyde-3-phosphate; DHAP, Dihydroxyacetone phosphate; F6P, Fructose-6-phosphate; G6P, Glucose-6-phosphate; PEP, phospho*enol*pyruvate; OAA, Oxaloacetate; Met. H4-folate, Methylene H4-folate; Gly, Glycine; Glu, Glutamine; Ser, Serine.

Proteins related to important metabolic processes such as photosynthesis, respiration, photorespiration and production of starch, amino acids and secondary metabolites were upregulated in the treatments with SL42 and SL48. This showed that the PGPR strains modulated major plant functions under optimal and salt-stressed conditions (Figure 7). Several proteins involved in seedling development, plant growth and stress responses were upregulated due to bacterial inoculation. Some of them are linked to phytohormone mediated pathways, suggesting that the bacteria influenced the signaling networks and modulated plant responses. A few key examples found in this study are discussed below.

### Rhizobacteria Upregulate Proteins Related to Molecular Functions, Nutrient Metabolism, and Photosynthesis

One of the important enzymes upregulated by SL42, SL48, and SL42 + SL48 under optimal conditions and linked to increased plant growth was asparagine synthetase 2 (ASN2). The enzyme is involved in asparagine synthesis and is essential for the regulation of nitrogen assimilation and reallocation within the plant *via* the phloem companion cells. It is predominantly expressed during darkness in vegetative leaves. It is important for primary metabolism, chlorophyll content, and biomass accumulation (Gaufichon et al., 2013). Hsp90 superfamily protein isoform X1 was upregulated in the treatments of SL42, SL48, and SL42 + SL48 under salt stress. Hsp90 is a molecular chaperone family essential for protein folding in the chloroplasts that are synthesized *de novo* or imported into the chloroplast mediated by the Toc/Tic complexes and cooperates with other chaperones. It is required for chloroplast development, specifically thylakoid formation within chloroplasts. Malfunction of Hsp90 has been shown to be lethal in transgenic Arabidopsis seeds, therefore it is essential for chloroplast biogenesis and embryogenesis (Oh et al., 2014). Another upregulated protein in the treatments of SL42, SL48, and SL42 + SL48 under salt stress was clathrin heavy chain (CHC), which is a subunit of clathrin, a major structural protein involved in the formation of clathrin-coated pits and clathrin-coated vesicles mediating endo- and exocytosis. One of the important functions of CHC is associated with stomatal movement linked to the expansion of guard cells. This in turn affects transpiration rate, gaseous exchange, and cell metabolism. Arabidopsis *chc* mutants showed defects in stomatal function and plant growth under water deficit. Endocytosis is also crucial for the polarized localization of PIN proteins (auxin transporters) and provides directional gradients for auxin distribution within the plant (Larson et al., 2017). Carbamoyl-phosphate synthase (CPS) is required for arginine biosynthesis, converting ornithine into citrulline. In higher plants, citrulline and arginine are essential for proper mesophyll development and reticulate venation in leaves (Molla-Morales et al., 2011). The enzyme is localized in the chloroplast and the large chain subunit was upregulated in the treatments SL42 + SL48, Bj + SL42, and Bj + SL48 and small chain by Bj + SL42 + SL48 under salt stress. Lipid transfer protein EARLI 1-like expressed only in the co-inoculation treatments of *B. japonicum* with SL42 and SL48. Upregulation of EARLI-1 improved seed germination, root elongation and reduced Na^+^ accumulation in leaves under salt stress. It is induced in embryonic tissues and young seedlings suggesting that it has a positive role in seed germination and early seedling development under high salinity stress (Xu et al., 2011). Proline-rich proteins (PRP) were upregulated in all the bacterial treatments under salt stress and also in SL42 + SL48, Bj + SL42 + SL48 under optimal conditions. PRP are major constituents of cell wall structure organization. They accumulated in the cell wall soluble fraction of common bean (*Phaseolus vulgaris*) in response to water deficit. It also accumulated in developing seedlings, specifically in the phloem tissues. It plays a role in plant morphogenesis and cell wall modification induced by osmotic stress (Battaglia et al., 2007). In another study, the soybean *GmPRP* gene showed distinct expression patterns in different organs from 2-week-old seedlings and was upregulated in response to abiotic and biotic stresses (He et al., 2002).

Carbonic anhydrases (CA) are the second most abundant protein cluster next to Rubisco in C3 plant leaves and catalyzes reversible hydration of CO_2_ to bicarbonate ion and proton. It is involved in CO_2_ diffusion and is closely associated with Rubisco activity. Its function is important for photosynthesis in response to drought stress. It also modulates stomatal conductance to promote water use efficiency, thereby helping plants adapt to water-deficit (Wang et al., 2016). It was upregulated by treatment with Bj + SL42 + SL48 under both conditions, supporting increased stress tolerance. Glutamine synthetase (GS) is upregulated by treatments with Bj + SL48 and Bj + SL42 + SL48 under optimal and salt stress conditions. It is a light-modulated enzyme targeted to leaf chloroplasts and mitochondria and primarily responsible for the reassimilation of ammonia generated by photorespiration in mitochondria, which is highly cytotoxic, and converts it to non-toxic glutamate in chloroplasts, and therefore, linked to plant growth (Taira et al., 2004). Lipoxygenases (LOX) are widely distributed in plants and catalyze hyperoxidation of polyunsaturated fatty acids containing a *cis*, *cis*-1,4-pentadiene structure to produce oxylipins. They play important physiological roles in seed germination, plant growth, nodule development, ripening, cell death, senescence, synthesis of ABA and jasmonic acid and responses to abiotic and biotic stresses. Soybean contains at least four distinct LOX isozymes in dry seeds and two isozymes in the hypocotyl/radicle region of the seedling stem. LOX act as vegetative storage proteins (VSPs), mobilize lipids and eliminate harmful ROS during rapid mobilization of nutrient reserves in germinating soybean seeds. LOXs were found in developing cotyledons, leaves and nodules. They also play crucial roles in abiotic stress responses by decreasing H_2_O_2_ accumulation and lipid peroxidation. Overexpression of *DkLOX3* (*Diospyros kaki* L. “Fupingjianshi”) in Arabidopsis was related to increased germination rate and upregulation of other stress-responsive genes under high drought and salinity stress conditions (Viswanath et al., 2020). The two major subfamilies, linoleate 13S-lipoxygenase and linoleate 9S-lipoxygenases, including seed linoleate 9S lipoxygenases, were upregulated by SL48 co-inoculation treatments under optimal and salt stress conditions.

### Proteins Involved in Phytohormone Mediated Responses Were Influenced by Rhizobacteria

Phytohormones are signaling molecules that regulate vital physiological processes and control plant responses to abiotic and biotic stresses including salinity stress (Waśkiewicz et al., 2016). Auxin is a key regulator of cell division, expansion, and differentiation in shoot and root meristems and plays crucial roles in plant development. Auxin binding protein abp19a-like (ABP19A) is an extracellular auxin receptor and binds to auxin. It is required for auxin responses in embryogenesis, and post-embryonic root growth and shoot development (Tromas et al., 2009). It was upregulated in all bacterial treatments, indicating that the bacteria play closely associated roles in auxin signaling, thereby promoting growth. The enzyme 1-aminocyclopropane-1-carboxylate oxidase (ACO) is involved in ethylene biosynthesis. Ethylene mediates the reversion of ABA-induced inhibition of seed germination *via* endosperm cap rupture. It also confers salinity tolerance by enhancement of Na/K homeostasis and accumulation of ascorbic acid through ethylene-mediated pathways (Linkies et al., 2009; Jiang et al., 2013). It was upregulated in the treatments of Bj + SL42 and Bj + SL48 under salt stress. Gibberellin-regulated protein 6 precursor (GASA6) is a small cysteine-rich peptide responsive to gibberellic acid (GA). It functions as an integrator in the downstream of GA signaling and regulates seed germination by promoting cell elongation at the embryonic axis. It takes part in redox reactions and decreases the accumulation of ROS in response to stress (Zhong et al., 2015). It was upregulated under salt stress by Bj + SL48 and Bj + SL42 + SL48 treatments. NADP dependent malic enzyme (NADP-ME) was upregulated in plants under salt stress with SL42 and SL48 inoculation and co-inoculation treatments with *B. japonicum*. It catalyzes the oxidative decarboxylation of malate to generate pyruvate, CO_2_, and NADPH. It plays functional roles in abscisic acid (ABA)-mediated signaling pathways related to seed development and osmotic stress. Treatment with ABA, NaCl and mannitol increased the accumulation of NADP-ME in Arabidopsis. The knockout *nadp-me1* mutants showed decreased seed viability, stomatal opening, and root growth. Hence, the enzyme participates during both seed germination and seedling growth stages. It is also essential to enhance tolerance of drought and saline conditions (Arias et al., 2018). Other proteins involved in the phytohormone-mediated responses that were upregulated by specific bacterial treatments are given in Table 3.

**TABLE 3:** Upregulated proteins involved in phytohormone-mediated responses.

Protein	Function*	Treatment	References
Abscicate beta-glucosyltransferase	Glycosylation of ABA and upregulated by ABA or drought stress.	Bj + SL42 (S)	Xu et al., 2002
Amidase 1 isoform X1	Involved in auxin biosynthesis. Converts indole-3-acetamide to indole-3-acetate.	Bj + SL48 (S)	Sanchez-Parra et al., 2014
Anthranilate synthase alpha subunit 1, chloroplastic	Part of a heterotetrameric complex that catalyzes the two-step biosynthesis of anthranilate, an intermediate in the biosynthesis of L-tryptophan. Plays an important regulatory role in auxin production *via* the tryptophan-dependent biosynthetic pathway.	SL42 + SL48 (S)	Stepanova et al., 2005
Aquaporin PIP2-7	Water channel required to facilitate the transport of water across cell membrane. Plays a predominant role in root water uptake process in conditions of reduced transpiration, and in osmotic fluid transport.	SL42 + SL48 (S)	Pou et al., 2016
Gamma-tocopherol methyltransferase	Biosynthesis of tocopherol. Protect the photosynthetic apparatus against oxidative stress.	Bj + SL42 + SL48 (S)	Bergmuller et al., 2003
Haem oxygenase	Key enzyme in the synthesis of the chromophore of the phytochrome family of plant photoreceptors. Plays a role in salt acclimation signaling. May affect the plastid-to-nucleus signaling pathway by perturbing tetrapyrrole synthesis.	Bj + SL42 + SL48 (S)	Gisk et al., 2010
IAA-amino acid hydrolase ILR1-like 4	Regulates amide-IAA hydrolysis and results in activation of auxin signaling.	SL42 (S)	Carranza et al., 2016
Peroxisomal 3-ketoacyl-CoA thiolase	Involved in long chain fatty-acid beta-oxidation prior to gluconeogenesis during germination and subsequent seedling growth.	Bj + SL42 + SL48	Germain et al., 2001
Protein PELPK1	Positive regulator of germination and plant growth.	Bj + SL42, SL42 + SL48	Rashid and Deyholos, 2011
Serine glyoxylate aminotransferase 3 isoform X1	Photorespiratory enzyme that catalyzes transamination reactions. Functions in asparagine metabolism. Involved in root development during seedling establishment after seed germination.	Bj + SL48	Zhang et al., 2013
Xanthoxin dehydrogenase	Generates abscisic aldehyde from xanthoxin, the last step of ABA biosynthetic pathway. Response to osmotic stress.	SL48 (S)	Gonzalez-Guzman et al., 2002

* Functional description of proteins was adapted from UniProt database. (S) Indicates treatments with salt stress. Bj, Bradyrhizobium japonicum 532C; SL42, Rhizobium sp. SL42; SL48, Hydrogenophaga sp. SL48.

## Conclusion

The analysis of leaf proteomic profile provided a comprehensive insight into the growth and salinity tolerance mechanisms of soybean plants modulated by the influence of rhizobacteria. These mechanisms are regulated by the inter-organismal communication, an intricate network of signaling pathways (Smith et al., 2015). In conclusion, soybean plants inoculated with *Rhizobium* sp. SL42 and *Hydrogenophaga* sp. SL48 enhanced vigor and salinity tolerance under growth chamber conditions. The bacteria triggered multiple signaling pathways that regulated growth and stress tolerance mechanisms, which in turn is a result of beneficial plant-microbe interaction. Nevertheless, plants co-inoculated with *Bradyrhizobium japonicum* 532C and the strains SL42 and SL48 exhibited higher growth-promoting and stress-alleviating mechanisms, suggesting compatible co-inoculation between the symbiont and the rhizobacteria. They play a crucial role in the development of soybean plants under stressful conditions and therefore could potentially be utilized as biostimulants to mitigate stress effects and boost crop productivity. This could ultimately lead to the crop improvement and salinity tolerance of soybean.

## Nomenclature

### Resource Identification Initiative

SAS 9.4 (Statistical Analysis System, RRID:SCR_008567).

Scaffold (Scaffold Proteome Software, RRID:SCR_014345).

Blast2GO (Blast2GO, RRID:SCR_005828).

The ProteomeExchange consortium (ProteomeXchange, RRID: SCR_004055).

## Data Availability Statement

The original contributions presented in the study are publicly available. This data can be found here: Data are available *via* ProteomeXchange with identifier PXD025596.

## Author Contributions

GI conducted the research, collected samples and data, and prepared the manuscript. SS and GI planned the experimental design and conducted proteomic analysis. SS and DS helped GI in editing the manuscript and providing feedback. All authors contributed to the article and approved the submitted version.

## Conflict of Interest

The authors declare that the research was conducted in the absence of any commercial or financial relationships that could be construed as a potential conflict of interest.

## Publisher’s Note

All claims expressed in this article are solely those of the authors and do not necessarily represent those of their affiliated organizations, or those of the publisher, the editors and the reviewers. Any product that may be evaluated in this article, or claim that may be made by its manufacturer, is not guaranteed or endorsed by the publisher.
