# Supplementary material for: Soybean Leaf Proteomic Profile Influenced by Rhizobacteria Under Optimal and Salt Stress Conditions
Source: Front Plant Sci. 2022 Mar 24;13:809906. doi: 10.3389/fpls.2022.809906 (PMC8987779; doi:10.3389/fpls.2022.809906)
Supplement: Supplementary file 1 [file Presentation_1.PDF]

Supplementary Figures and Tables

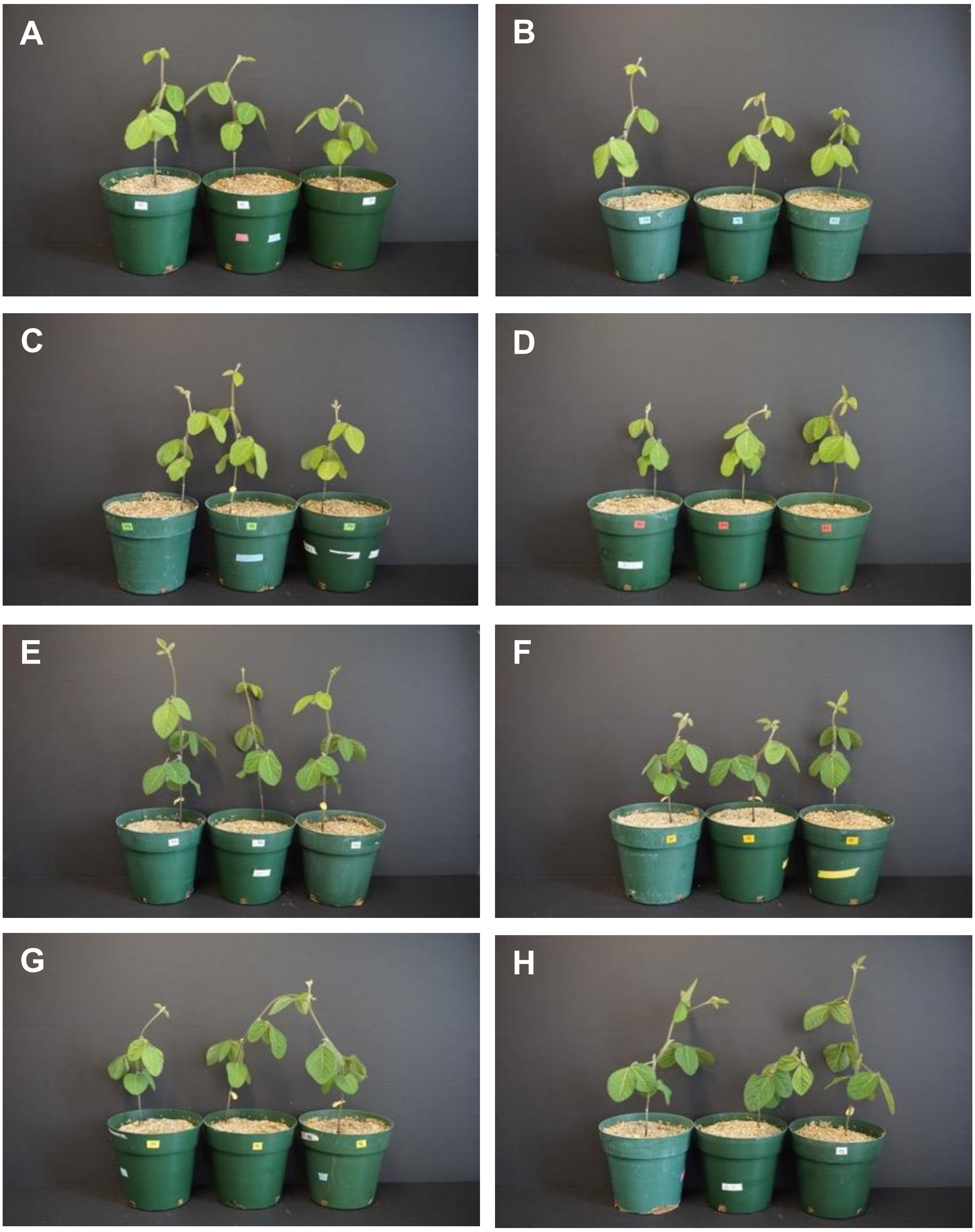

Supplemetary Figure S1. Soybean plants at 28th DAP grown in controlled environment under optimal and salt-stressed conditions. The treatments are **(A)** control **(B)** SL42 **(C)** SL48 **(D)** SL42+SL48 **(E)** Bj **(F)** Bj+SL42 **(G)** Bj+SL48 **(H)** Bj+SL42+SL48. The seeds were treated with 10 mM $MgSO_4$ or bacterized with the *Rhizobium* sp. SL42, *Hydrogenophaga* sp. SL48 or co-inoculated and (B) Seeds were bacterized with *Bradyrhizobium japonicum* (Bj) as control or the strains were co-inoculated with Bj.

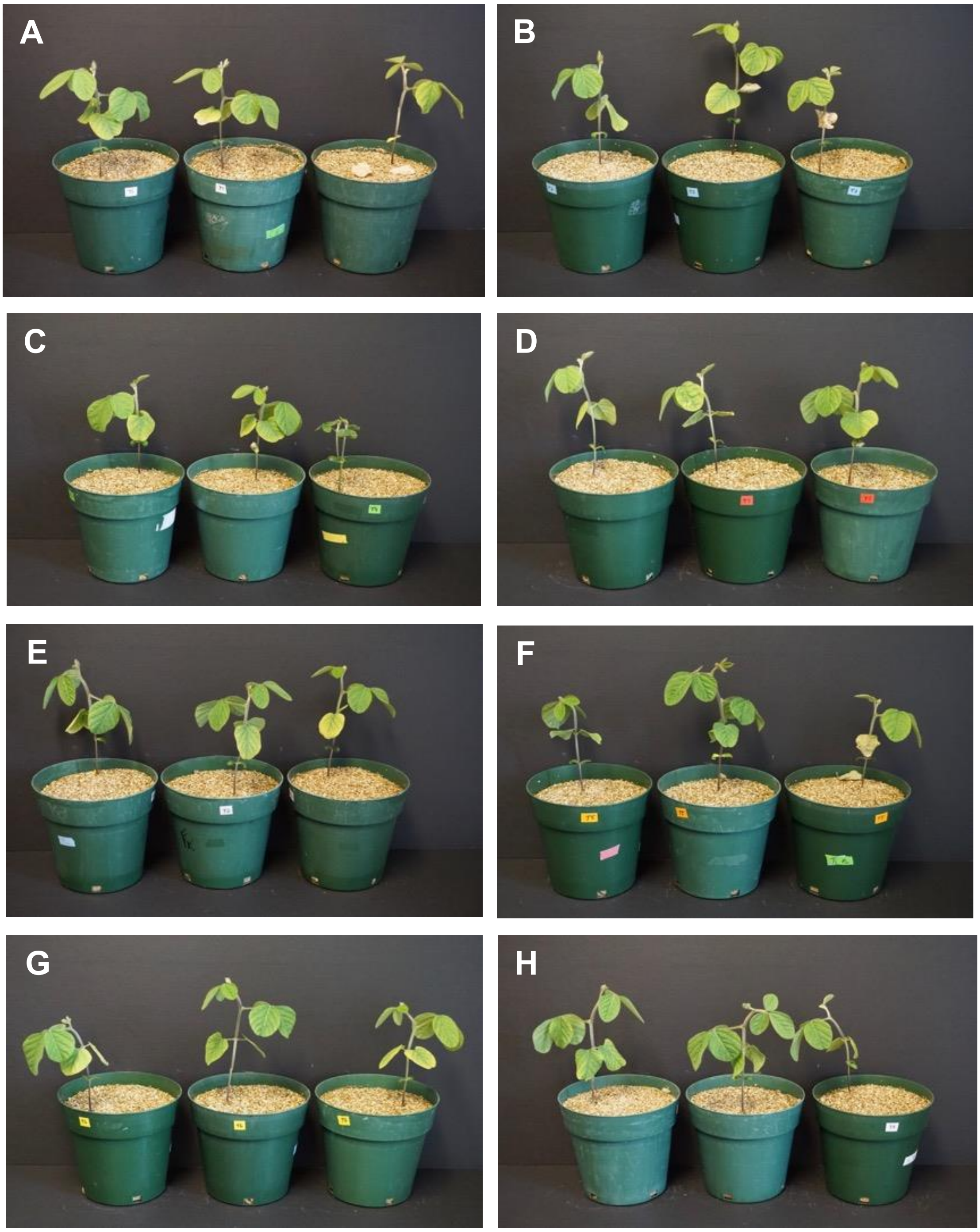

Supplemetary Figure S2. (cont.) Soybean plants at 28th DAP grown in controlled environment under optimal and salt-stressed conditions. The treatments are **(A)** control **(B)** SL42 **(C)** SL48 **(D)** SL42+SL48 **(E)** Bj **(F)** Bj+SL42 **(G)** Bj+SL48 **(H)** Bj+SL42+SL48. The seeds were treated with 10 mM $MgSO_4$ or bacterized with the *Rhizobium* sp. SL42, *Hydrogenophaga* sp. SL48 or co-inoculated and (B) Seeds were bacterized with *Bradyrhizobium japonicum* (Bj) as control or the strains were co-inoculated with Bj.

Supplementary Table S2. Elemental analysis of major nutrients of soybean shoot tissue at 28th DAP under optimal and salt stress conditions.

	Nitrogen (mg. g^{-1})		Phosphorous (mg. g^{-1})		Calcium (mg. g^{-1})	
p-value	0.9257		0.0082		< 0.0001	
**Treatments**	Optimal	Salt	Optimal	Salt	Optimal	Salt
Ctrl	15.4 ±2.4^a	18.3 ±0.9^a	1.0 ±0.1^b	1.5 ±0.2^a	7.2 ±0.2^a	4.8 ±0.4^b
SL42	17.3 ±2.3^a	18.5 ±1.6^a	1.2 ±0.2^{ab}	1.6 ±0.2^a	6.7 ±0.9^a	4.9 ±0.4^b
SL48	15.6 ±2.6^a	17.9 ±1.4^a	1.0 ±0.1^b	1.5 ±0.2^a	7.7 ±0.6^a	4.9 ±0.2^b
SL42+SL48	17.9 ±2.5^a	17.6 ±1.4^a	1.0 ±0.1^b	1.5 ±0.1^a	7.1 ±0.2^a	4.5 ±0.4^b
p-value	0.8148		0.0005		< 0.0001	
Bj	20.1 ±1.0^a	20.4 ±0.8^a	0.8 ±0.1^b	1.4 ±0.1^a	6.6 ±0.4^a	4.8 ±0.2^b
Bj+SL42	21.5 ±1.0^a	22.0 ±1.6^a	0.9 ±0.1^b	1.5 ±0.2^a	6.7 ±0.5^a	4.6 ±0.2^b
Bj+SL48	21.4 ±0.6^a	22.1 ±1.2^a	0.9 ±0.1^b	1.5 ±0.2^a	6.6 ±0.4^a	4.6 ±0.2^b
Bj+SL42+ SL48	21.2 ±0.8^a	21.7 ±0.7^a	0.9 ±0.1^b	1.4 ±0.2^a	6.9 ±0.3^a	4.6 ±0.2^b
	Potassium (mg. g^{-1})		Sodium (mg. g^{-1})		K:Na	
p-value	< 0.0001		< 0.0001			
**Treatments**	Optimal	Salt	Optimal	Salt	Optimal	Salt
Ctrl	10.5 ±1.6^b	21.3 ±1.6^a	0.05 ±0.0^b	0.3 ±0.0^a	213.42	68.89
SL42	11.4 ±1.3^b	23.2 ±2.4^a	0.06 ±0.0^b	0.3 ±0.0^a	203.52	85.63
SL48	11.3 ±1.0^b	23.5 ±1.7^a	0.06 ±0.0^b	0.3 ±0.0^a	186.71	81.62
SL42+SL48	10.6 ±1.4^b	21.4 ±1.2^a	0.04 ±0.0^b	0.3 ±0.0^a	246.65	82.20
p-value	< 0.0001		< 0.0001			
Bj	9.8 ±1.3^b	22.5 ±2.8^a	0.08 ±0.1^b	0.4 ±0.1^a	116.98	57.96
Bj+SL42	10.1 ±1.1^b	20.1 ±1.4^a	0.07 ±0.0^b	0.3 ±0.1^a	151.88	63.27
Bj+SL48	10.7 ±1.1^b	22.5 ±2.4^a	0.06 ±0.0^b	0.4 ±0.1^a	187.87	53.77
Bj+SL42+ SL48	10.1 ±1.1^b	20.9 ±1.6^a	0.05 ±0.0^b	0.4 ±0.0^a	199.81	59.83

Values represent mean ± SE (n=4) and values that share the same letters are not significantly different ($\alpha = 0.05$). Bj – *Bradyrhizobium japonicum* 532C, SL42 – *Rhizobium* sp. SL42, SL48 – *Hydrogenophaga* sp. SL48.

Supplementary Table S3. Fold change of selected proteins that were commonly upregulated by the treatments SL42, SL48 and SL42+SL48 relative to control under optimal condition.

Accession Number	Protein	SL42	SL48	SL42+ SL48
NP_001235721.1 [5]	Cluster of asparagine synthetase 2	4.5	13	9.5
NP_001241264.1 [2]	Cluster of glucose-6-phosphate dehydrogenase	3.3		3.3
XP_006584638.1 [3]	Cluster of PREDICTED: glutathione S-transferase GST 9 isoform X1	1.4	1.4	1.4
XP_014618589.1 [2]	Cluster of S-formylglutathione hydrolase		3.2	2.6
YP_538812.1	NADH dehydrogenase subunit 7 (chloroplast)	1.5		1.5
XP_003550402.1	PREDICTED: thioredoxin H1	1.3	1.3	
XP_003548656.1	PREDICTED: UDP-glucose flavonoid 3-O-glucosyltransferase 7-like		1.7	1.9
XP_003523219.1	prolyl endopeptidase	1.4	1.5	1.8
NP_001241137.1 (+1)	ribulose bisphosphate carboxylase small chain 4	1.2	1.1	1.3
NP_001240857.1	soyasaponin III rhamnosyltransferase		1.3	1.5

Values represent fold change of quantitative spectra relative to control ($P \leq 0.05$; n=3).
SL42 – *Rhizobium* sp. SL42, SL48 – *Hydrogenophaga* sp. SL48

Supplementary Table S4. Fold change of selected proteins that were commonly upregulated by the treatments SL42, SL48 and SL42+SL48 relative to control under salt stress.

Accession Number	Protein	SL42	SL48	SL42+ SL48
XP_003522764.1 (+1)	argininosuccinate lyase, chloroplastic isoform X1	2		2
NP_001235244.1 [2]	Cluster of alpha-amylase inhibitor/lipid transfer/seed storage family protein precursor		5.3	4
NP_001236999.1 [5]	Cluster of clathrin heavy chain	1.3		1.3
XP_003537859.1 [3]	Cluster of coatomer subunit delta	2.9	2.5	
XP_003522620.1 [4]	Cluster of DEAD-box ATP-dependent RNA helicase 3, chloroplastic		1.8	2
XP_003555893.1 [3]	Cluster of dihydrolipoyllysine-residue acetyltransferase component 4 of pyruvate dehydrogenase complex, chloroplastic isoform X2		2.3	2.4
NP_001238484.1 [3]	Cluster of glyceraldehyde-3-phosphate dehydrogenase A subunit	1.3	1.4	1.3
XP_003550270.1 [6]	Cluster of glycine dehydrogenase (decarboxylating), mitochondrial		1.2	1.3
XP_006601356.1 [4]	Cluster of HSP90 superfamily protein isoform X1	1.4	1.4	1.3
XP_003537948.1 [2]	Cluster of linoleate 13S-lipoxygenase 2-1, chloroplastic		1.8	1.8

Accession Number	Protein	Bj+SL42	Bj+SL48	Bj+SL42+SL48
XP_003538388.1 [2]	Cluster of NADH dehydrogenase [ubiquinone] iron-sulfur protein 1, mitochondrial		1.6	1.2
XP_003534202.1 [2]	Cluster of peroxisomal fatty acid beta-oxidation multifunctional protein AIM1	1.5	1.4	
NP_001235098.1 [2]	Cluster of proline-rich protein precursor	10	7	5.7
XP_003546552.1 [5]	Cluster of S-adenosylmethionine synthase		1.2	1.3
XP_003517160.1 [3]	Cluster of SKP1-like protein 1A isoform X1		2.2	1.9
XP_003545030.1 (+1)	endoplasmin homolog isoform X1	1.4	1.4	1.3
XP_003533844.1 (+1)	photosystem I reaction center subunit psaK	2.3	2.7	3.2
XP_003547722.1	PREDICTED: auxin-binding protein ABP19a-like	2.3	2.1	1.9
XP_006595483.1	PREDICTED: peroxisomal citrate synthase isoform X1	4.2	4.5	
XP_003537582.1	succinate dehydrogenase [ubiquinone] flavoprotein subunit 1, mitochondrial		1.6	1.7
XP_014634634.1 (+1)	topless-related protein 1 isoform X1		2.2	1.8
XP_006599577.1 (+1)	tripeptidyl-peptidase 2 isoform X1		2.2	2.4

Values represent fold change of quantitative spectra relative to control ($P \leq 0.05$; n=3). SL42 – *Rhizobium* sp. SL42, SL48 – *Hydrogenophaga* sp. SL48

Supplementary Table S5. Fold change of selected proteins that were commonly upregulated by the treatments Bj+SL42, Bj+SL48 and Bj+SL42+SL48 relative to Bj (control) under optimal condition.

Accession Number	Protein	Bj+ SL42	Bj+ SL48	Bj+SL42+SL48
XP_003556239.1 (+2)	ATPase ARSA1		1.9	1.8
NP_001304442.1 [3]	Cluster of adenosylhomocysteinase-like		1.3	1.3
XP_003520429.1 [2]	Cluster of bifunctional monothiol glutaredoxin-S16, chloroplastic		2	1.9
NP_001240021.1 [4]	Cluster of catalase		1.2	1.3
XP_003528751.1 [3]	Cluster of chlorophyll a-b binding protein		1.4	1.3
NP_001238484.1 [3]	Cluster of glyceraldehyde-3-phosphate dehydrogenase A subunit	1.2	1.8	1.6
XP_003544533.1 [4]	Cluster of glycine dehydrogenase (decarboxylating), mitochondrial	1.2	1.3	1.2
NP_001237637.1 [2]	Cluster of isoflavone reductase homolog 2	3.1	3.5	3.6
XP_003534511.1 [2]	Cluster of isopentenyl-diphosphate Delta-isomerase I	1.7	2	2.5
XP_003532236.1 [2]	Cluster of kunitz-type trypsin inhibitor KTI1-like	1.6	1.8	

Accession Number	Protein	Bj+ SL42	Bj+ SL48	Bj+SL42+SL48
NP_001238692.1 [5]	Cluster of linoleate 9S-lipoxygenase-4		1.2	1.2
XP_003545754.1 [2]	Cluster of peptide methionine sulfoxide reductase B5	6.5	6.5	6.2
XP_014622135.1 [2]	Cluster of PREDICTED: multicystatin	2.1	2.3	1.9
NP_001235098.1 [2]	Cluster of proline-rich protein precursor		19	6.3
XP_003546552.1 [7]	Cluster of S-adenosylmethionine synthase	1.2	1.4	1.3
XP_003546674.1 [4]	Cluster of soyasapogenol B glucuronide galactosyltransferase-like	1.4	1.7	1.4
XP_003540156.3	ferredoxin-A	2.1	2.9	3.7
NP_001237784.1	glutamine synthetase precursor		1.2	1.2
XP_003540396.1	ketol-acid reductoisomerase, chloroplastic	1.3	1.3	
NP_001236605.1	KS-type dehydrin SLTI629		4.1	6
XP_003533844.1 (+1)	photosystem I reaction center subunit psaK	1.7	2.8	2.9
XP_006594327.2	polyphenol oxidase A1, chloroplastic	2.4	2.5	2.4
XP_003547722.1	PREDICTED: auxin-binding protein ABP19a-like	1.6	2.3	1.8
XP_003548656.1	PREDICTED: UDP-glucose flavonoid 3-O-glucosyltransferase 7-like	1.7	1.9	1.6
XP_003536005.1	protoporphyrinogen oxidase 1, chloroplastic		1.7	1.5
NP_001235314.1 (+2)	ribulose bisphosphate carboxylase small chain 1	1.3	1.4	1.3
NP_001240857.1	soyasaponin III rhamnosyltransferase	1.4	1.5	
XP_006595826.2	subtilisin-like protease Glyma18g48580 isoform X1	2.9	2.3	2.4
XP_003537582.1	succinate dehydrogenase [ubiquinone] flavoprotein subunit 1, mitochondrial		1.8	1.8
XP_003538169.1	superoxide dismutase [Cu-Zn], chloroplastic	2.5	2.3	2.4

Values represent fold change of quantitative spectra relative to control ($P \leq 0.05$; n=3). Bj – *Bradyrhizobium japonicum* 532C, SL42 – *Rhizobium* sp. SL42, SL48 – *Hydrogenophaga* sp. SL48

Supplementary Table S6. Fold change of selected proteins that were commonly upregulated by the treatments Bj+SL42, Bj+SL48 and Bj+SL42+SL48 relative to Bj (control) under salt stress.

Accession Number	Protein	Bj+ SL42	Bj+ SL48	Bj+SL42+SL48
XP_003519448.1 [3]	Cluster of 1-aminocyclopropane-1-carboxylate oxidase	1.6	1.7	
XP_003538537.1 [2]	Cluster of 15-cis-phytoene desaturase, chloroplastic/chromoplastic	1.6	2.2	1.6
XP_003554099.1 [2]	Cluster of 4-alpha-glucanotransferase DPE2		1.4	1.4
XP_003543388.1 [3]	Cluster of aconitate hydratase, cytoplasmic	1.3	1.4	
XP_003535935.1 [2]	Cluster of calvin cycle protein CP12-2		1.4	1.8
XP_003542003.2 [2]	Cluster of carbamoyl-phosphate synthase large chain, chloroplastic	1.6	1.6	
NP_001240021.1 [4]	Cluster of catalase	1.3	1.3	1.2
XP_003522620.1 [4]	Cluster of DEAD-box ATP-dependent RNA helicase 56	1.5		1.5
NP_001236470.1 [6]	Cluster of gibberellin-regulated protein 6 precursor		1.6	1.2
XP_014620707.1 [2]	Cluster of glutamine synthetase precursor isoform X1		1.3	1.3
NP_001238484.1 [3]	Cluster of glyceraldehyde-3-phosphate dehydrogenase A subunit	1.3	1.1	
XP_003550270.1 [4]	Cluster of glycine dehydrogenase (decarboxylating), mitochondrial	1.2	1.1	1.2
XP_003516804.1 [8]	Cluster of NADP-dependent malic enzyme	1.4	1.3	1.2
XP_003530536.2 [2]	Cluster of probable 3-hydroxyisobutyrate dehydrogenase-like 1, mitochondrial	2.5	2	
NP_001235098.1 [2]	Cluster of proline-rich protein precursor	3.3	2.6	1.8
NP_001240245.1 [5]	Cluster of ribulose bisphosphate carboxylase/oxygenase activase	1.1	1.2	1.1
XP_006596615.1	cucumisin	1.6	1.8	1.9
XP_014633122.1	glucose-6-phosphate 1-dehydrogenase		1.7	1.5
XP_003517945.1	heme-binding protein 2		1.3	1.6
XP_003525916.1	MFP1 attachment factor 1	4.3	4.3	
XP_003553172.1 (+1)	NAD(P)H-quinone oxidoreductase subunit N, chloroplastic	6	6.5	5
XP_003533844.1 (+1)	photosystem I reaction center subunit psaK	1.6	1.4	1.5
YP_538794.1	photosystem II protein H		1.3	1.9
NP_001239837.1	polygalacturonase inhibitor 1-like protein precursor	3.5	4.5	2.9
XP_003548656.1	PREDICTED: UDP-glucose flavonoid 3-O-glucosyltransferase 7-like	2.4	2.9	2.6

Accession Number	Protein	Bj+ SL42	Bj+ SL48	Bj+SL42+SL48
XP_003516432.1	probable UDP-arabinopyranose mutase 1		1.5	1.4
XP_014633159.1 (+1)	protein TOPLESS		2.2	2.2
XP_003555218.1	protein transport protein Sec24-like At4g32640		3.2	4.6
NP_001241137.1	ribulose bisphosphate carboxylase small chain 4	1.4	1.5	1.2
YP_538747.1	ribulose-1,5-bisphosphate carboxylase/oxygenase large subunit	1.2	1.2	1.2
XP_006604796.1	topless-related protein 3		4.3	3.3
XP_003549747.1 (+3)	topless-related protein 4 isoform X2		3.8	3
XP_003536615.2	UDP-glycosyltransferase 84B2	3.4	3.2	3.2
XP_003554142.1	UDP-sulfoquinovose synthase, chloroplastic	2		1.7

Values represent fold change of quantitative spectra relative to control ($P \leq 0.05$; n=3). Bj – *Bradyrhizobium japonicum* 532C, SL42 – *Rhizobium* sp. SL42, SL48 – *Hydrogenophaga* sp. SL48

Supplementary Table S7. Proteins that were specifically upregulated by treatments SL42, SL48 and SL42+SL48 relative to control under optimal condition.

<table>
<tr><th>SL42</th><th>SL42+SL48</th></tr>
<tr><td>• Cluster of matrix metalloproteinase precursor
• isoflavone reductase-like protein</td><td rowspan="3">• Cluster of aconitate hydratase 1
• Cluster of adenosylhomocysteinase
• Cluster of catalase
• Cluster of chlorophyll a-b binding protein P4, chloroplastic
• Cluster of fumarate hydratase 1, mitochondrial
• Cluster of glyceraldehyde-3-phosphate dehydrogenase A subunit
• Cluster of glycine dehydrogenase (decarboxylating), mitochondrial
• Cluster of linoleate 9S-lipoxygenase-4
• Cluster of peroxisomal (S)-2-hydroxy-acid oxidase GLO1-like
• Cluster of phosphoenolpyruvate carboxylase
• indole-3-glycerol phosphate synthase, chloroplastic
• NAD(P)H-quinone oxidoreductase subunit N, chloroplastic
• photosystem I reaction center subunit psaK, chloroplastic
• protein PELPK1
• ribulose-1,5 bisphosphate carboxylase/oxygenase large subunit N-methyltransferase, chloroplastic
• soyasapogenol B glucuronide galactosyltransferase-like</td></tr>
<tr><td>SL48</td></tr>
<tr><td>• 26S proteasome regulatory subunit 4 homolog A
• Cluster of clathrin heavy chain 2
• stress-induced protein SAM22
• UDP-glucuronic acid decarboxylase 2</td></tr>
</table>

Fold change ≥ 1.2 (P ≤ 0.05; n=3). SL42 – *Rhizobium* sp. SL42, SL48 – *Hydrogenophaga* sp. SL48

Supplementary Table S8. Proteins that were specifically upregulated by treatments SL42, SL48 and SL42+SL48 relative to control under salt stress.

<table>
<tr><th>SL42</th><th>SL42+SL48</th></tr>
<tr><td>• 12-oxophytodienoate reductase 3
• Cluster of ABC transporter C family member 4
• Cluster of alpha-glucan water dikinase, chloroplastic isoform X1
• Cluster of calnexin homolog precursor
• Cluster of chaperonin CPN60-2, mitochondrial
• Cluster of citrate synthase, glyoxysomal
• Cluster of IAA-amino acid hydrolase ILR1-like 4
• osmotin-like protein
• PREDICTED: ferredoxin-A-like
• protein ROOT HAIR DEFECTIVE 3 homolog 2</td>
<td rowspan="3">• aquaporin PIP2-7
• Cluster of aconitate hydratase, cytoplasmic
• Cluster of carbamoyl-phosphate synthase large chain, chloroplastic
• Cluster of chlorophyll a/b-binding protein
• Cluster of diphosphomevalonate decarboxylase MVD2, peroxisomal
• Cluster of magnesium-protoporphyrin IX monomethyl ester [oxidative] cyclase, chloroplastic
• Cluster of plastidial pyruvate kinase 2
• Cluster of PREDICTED: zinc finger BED domain-containing protein DAYSLEEPER-like
• Cluster of protochlorophyllide reductase, chloroplastic
• Cluster of subtilisin-like protease SBT1.6
• D-3-phosphoglycerate dehydrogenase 2, chloroplastic
• fatty acid hydroperoxide lyase, chloroplastic
• heat shock 70 kDa protein 14 isoform X1
• KS-type dehydrin SLTI629
• NADH dehydrogenase subunit 7 (chloroplast)
• pyruvate dehydrogenase E1 component subunit beta-3, chloroplastic
• pyruvate kinase 1, cytosolic isoform X1
• ribulose-1,5-bisphosphate carboxylase/oxygenase large subunit (chloroplast)
• soyasaponin III rhamnosyltransferase
• succinate dehydrogenase [ubiquinone] iron-sulfur subunit 2, mitochondrial</td></tr>
<tr><th>SL48</th></tr>
<tr><td>• ATP synthase CF1 beta subunit (chloroplast)
• chlorophyll a-b binding protein 3, chloroplastic
• Cluster of cytosolic chaperonin
• Cluster of gamma carbonic anhydrase 1, mitochondrial
• Cluster of heat shock 70 kDa protein 14
• Cluster of phosphoglucomutase, chloroplastic
• nifU-like protein 4, mitochondrial
• pullulanase 1, chloroplastic
• succinate--CoA ligase [ADP-forming] subunit alpha, mitochondrial
• xanthoxin dehydrogenase</td></tr>
</table>

Fold change ≥ 1.2 (P ≤ 0.05; n=3). SL42 – *Rhizobium* sp. SL42, SL48 – *Hydrogenophaga* sp. SL48

Supplementary Table S9. Proteins that were specifically upregulated by treatments Bj+SL42, Bj+SL48 and Bj+SL42+SL48 relative to Bj (control) under optimal condition.

<table>
<tr><th>Bj+SL42</th><th>Bj+ SL42+SL48</th></tr>
<tr><td>• Cluster of isoamylase 3, chloroplastic isoform X1
• Cluster of ribosomal protein L11 family protein
• Cluster of subtilisin-like protease Glyma18g48580
• kunitz family trypsin and protease inhibitor protein precursor
• peptide methionine sulfoxide reductase B5
• PREDICTED: peptidyl-prolyl cis-trans isomerase 1-like
• protein PELPK1
• succinate dehydrogenase [ubiquinone] iron-sulfur subunit 2, mitochondrial</td><td rowspan="3">• carbonic anhydrase 2
• chaperonin CPN60-like 2, mitochondrial
• Cluster of carbamoyl-phosphate synthase large chain, chloroplastic
• Cluster of PREDICTED: zinc finger BED domain-containing protein DAYSLEEPER-like
• Cluster of pyrophosphate-energized vacuolar membrane proton pump
• Cluster of transketolase, chloroplastic
• glutathione S-transferase L3
• NAD(P)H-quinone oxidoreductase subunit N, chloroplastic
• peroxisomal 3-ketoacyl-CoA thiolase
• protochlorophyllide reductase, chloroplastic
• putative plastocyanin
• ribulose-1,5-bisphosphate carboxylase/oxygenase large subunit</td></tr>
<tr><td>Bj+SL48</td></tr>
<tr><td>• 15-cis-phytoene desaturase, chloroplastic/chromoplastic
• aquaporin PIP2-10
• Cluster of DEAD-box ATP-dependent RNA helicase 3, chloroplastic
• Cluster of linoleate 13S-lipoxygenase 2-1, chloroplastic
• Cluster of serine glyoxylate aminotransferase 3 isoform X1
• gamma-glutamyl hydrolase precursor
• indole-3-glycerol phosphate synthase, chloroplastic
• iron-superoxide dismutase
• malonyl-CoA:isoflavone 7-O-glucoside-6"-O-malonyltransferase
• probable carboxylesterase 2
• ribulose bisphosphate carboxylase/oxygenase activase
• UDP-glucosyl transferase 73B2</td></tr>
</table>

Fold change ≥ 1.2 ($P \leq 0.05$; n=3). Bj – *Bradyrhizobium japonicum* 532C, SL42 – *Rhizobium* sp. SL42, SL48 – *Hydrogenophaga* sp. SL48

Supplementary Table S10. Proteins that were specifically upregulated by treatments Bj+SL42, Bj+SL48 and Bj+SL42+SL48 relative to Bj (control) under salt stress.

<table>
<tr><th>Bj+SL42</th><th>Bj +SL48</th></tr>
<tr><td>• 12-oxophytodienoate reductase 3
• abscisate beta-glucosyltransferase
• caffeic acid 3-O-methyltransferase-like
• Cluster of ferritin
• Cluster of iron-superoxide dismutase
• Cluster of pyrophosphate--fructose 6-phosphate 1-phosphotransferase subunit alpha
• ferredoxin-A
• harpin binding protein 1
• putative glucose-6-phosphate 1-epimerase
• ruBisCO large subunit-binding protein subunit beta, chloroplastic
• stress-induced protein SAM22
• superoxide dismutase [Fe], chloroplastic precursor</td><td rowspan="3">• alpha-amylase inhibitor/lipid transfer/seed storage family protein precursor
• amidase 1 isoform X1
• Cluster of adenylosuccinate synthetase 2, chloroplastic
• Cluster of peroxisomal glycolate oxidase isoform X1
• Cluster of pyruvate decarboxylase 2
• glutamate decarboxylase
• lipoxygenase
• peroxisomal (S)-2-hydroxy-acid oxidase GLO1-like
• phi class glutathione S-transferase
• phosphoenolpyruvate carboxylase 4
• PREDICTED: auxin-binding protein ABP19a-like
• probable glutathione S-transferase
• soyasapogenol B glucuronide galactosyltransferase
• soyasaponin III rhamnosyltransferase
• trifunctional UDP-glucose 4,6-dehydratase/UDP-4-keto-6-deoxy-D-glucose 3,5-epimerase/UDP-4-keto-L-rhamnose-reductase RHM1
• seed linoleate 9S-lipoxygenase</td></tr>
<tr><td>Bj+ SL42+SL48</td></tr>
<tr><td>• carbamoyl-phosphate synthase small chain, chloroplastic
• carbonic anhydrase 2
• Cluster of ATP synthase subunit b', chloroplastic
• Cluster of gamma-tocopherol methyltransferase
• Cluster of PREDICTED: phosphoglycerate kinase, cytosolic
• Cluster of stromal 70 kDa heat shock-related protein, chloroplastic
• granule bound starch synthase Ia
• NADH dehydrogenase subunit 7 (chloroplast)
• phosphoglycerate kinase, cytosolic
• photosystem I subunit VII (chloroplast)
• PREDICTED: thioredoxin H1
• thioredoxin M1, chloroplastic</td></tr>
</table>

Fold change ≥ 1.2 ($P \le 0.05$; n=3). Bj – *Bradyrhizobium japonicum* 532C, SL42 – *Rhizobium* sp. SL42, SL48 – *Hydrogenophaga* sp. SL48

Functional classification of proteins based on GO categories

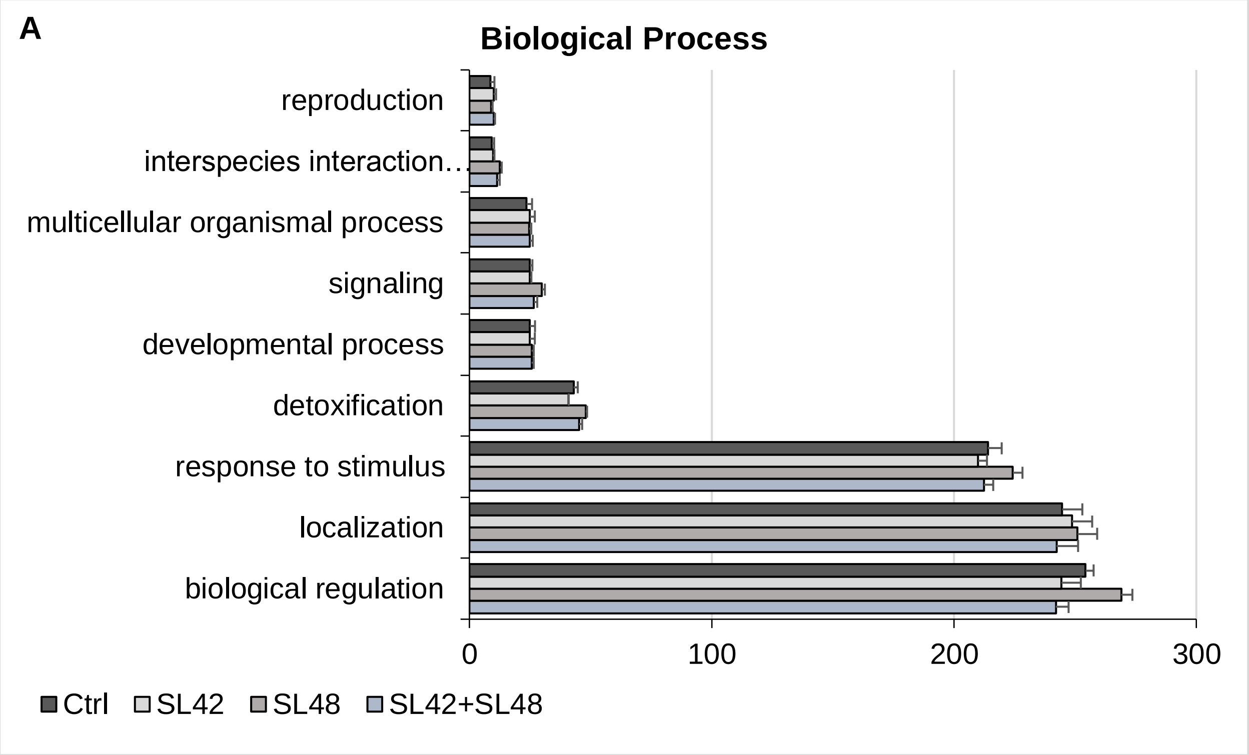

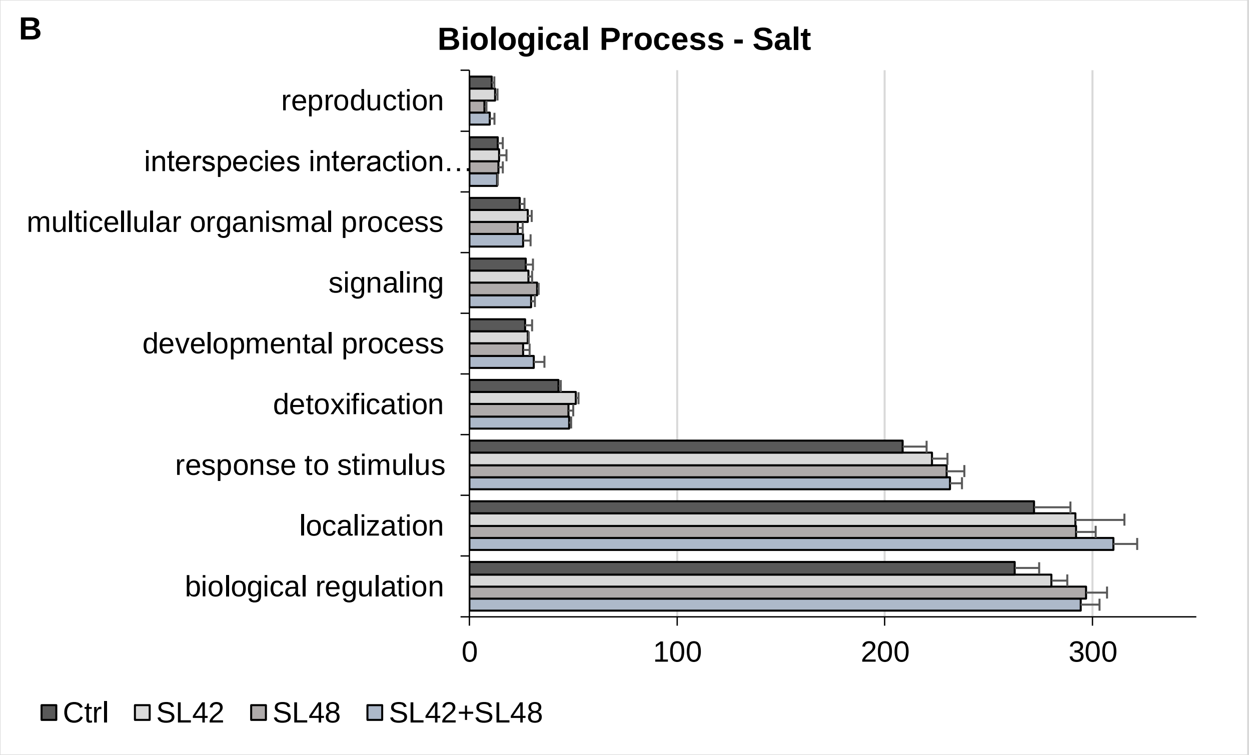

Supplementary Figure S2. Number of sequences involved in the biological processes of the soybean leaf proteome. **(A)** The seeds were treated with 10 mM $MgSO_4$ or bacterized with the strains *Rhizobium* sp. SL42, *Hydrogenophaga* sp. SL48 or co-inoculated under optimal and **(B)** under salt stress conditions. Values represent mean ± SE (n=3).

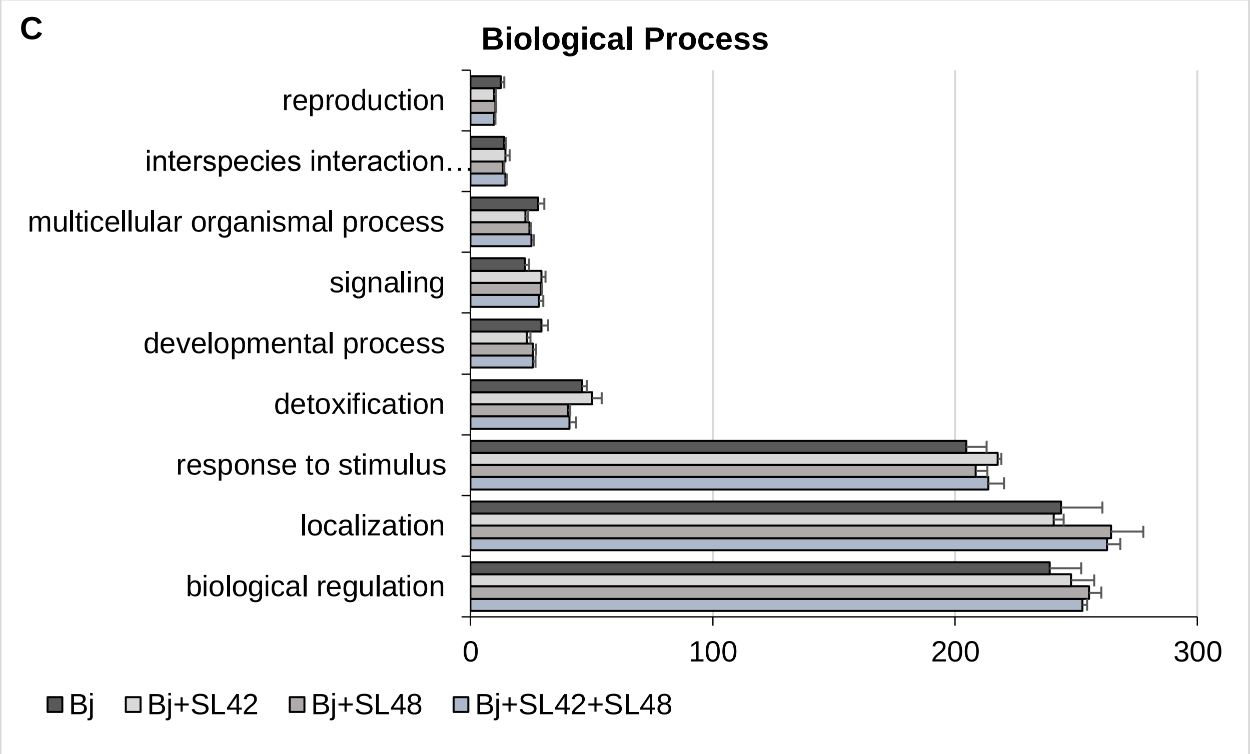

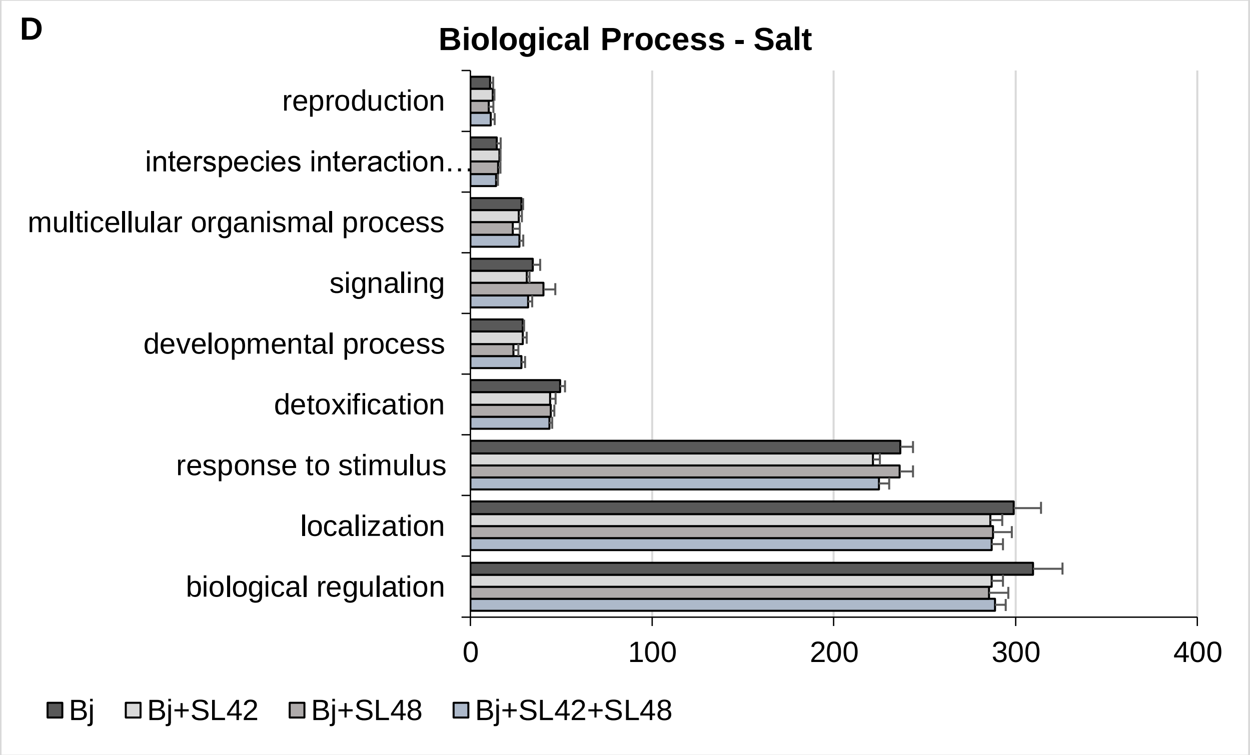

Supplementary Figure S2. (cont.) Number of sequences involved in the biological processes of the soybean leaf proteome. **(C)** seeds were bacterized with *Bradyrhizobium japonicum* (Bj) as control or the strains *Rhizobium* sp. SL42, *Hydrogenophaga* sp. SL48 co-inoculated with Bj under optimal and **(D)** under salt stress conditions. Values represent mean ± SE (n=3).

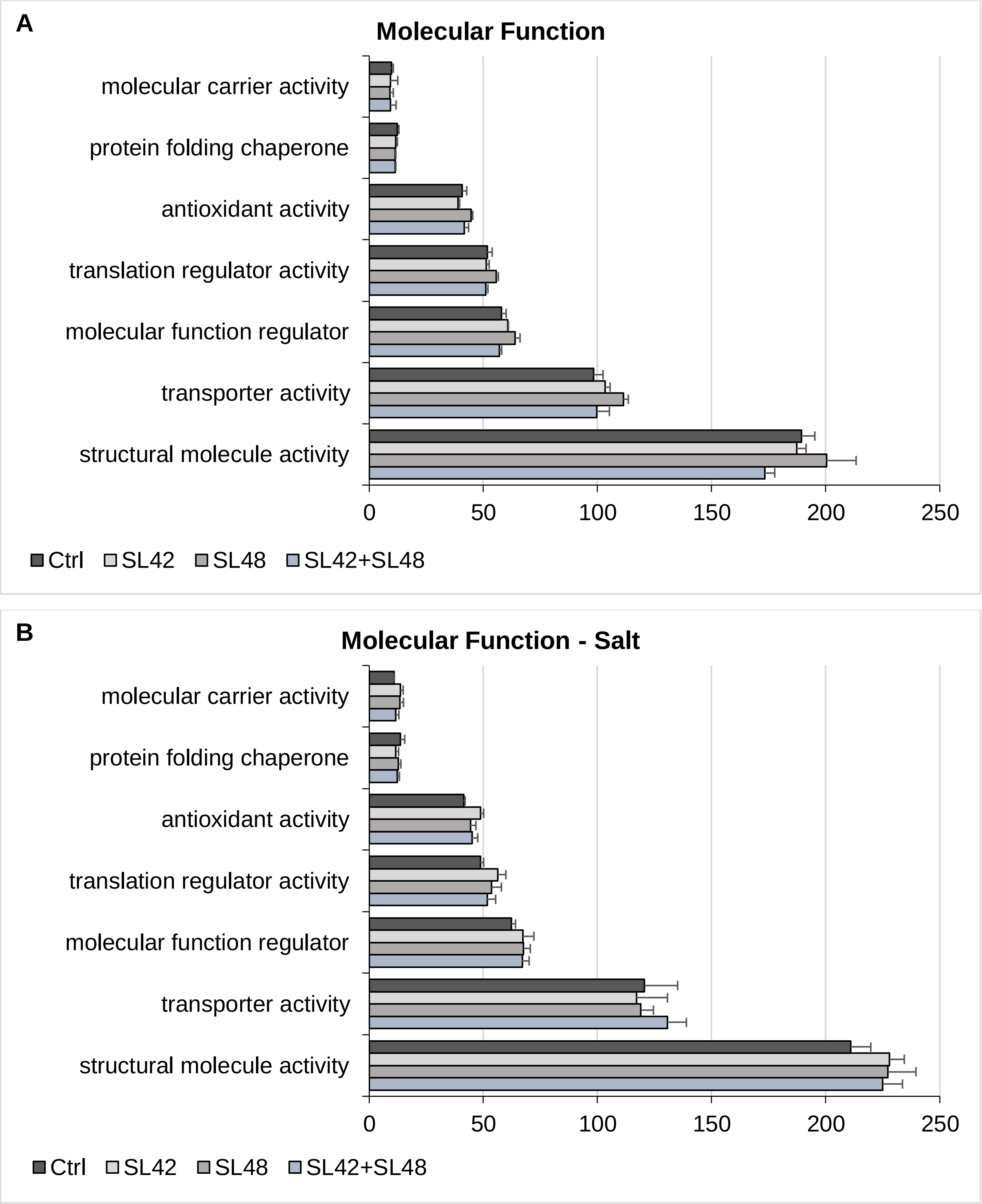

Supplementary Figure S3. Number of sequences involved in the molecular functions of the soybean leaf proteome. **(A)** The seeds were treated with 10 mM $MgSO_4$ or bacterized with the strains *Rhizobium* sp. SL42, *Hydrogenophaga* sp. SL48 or co-inoculated under optimal and **(B)** under salt stress conditions. Values represent mean ± SE (n=3).

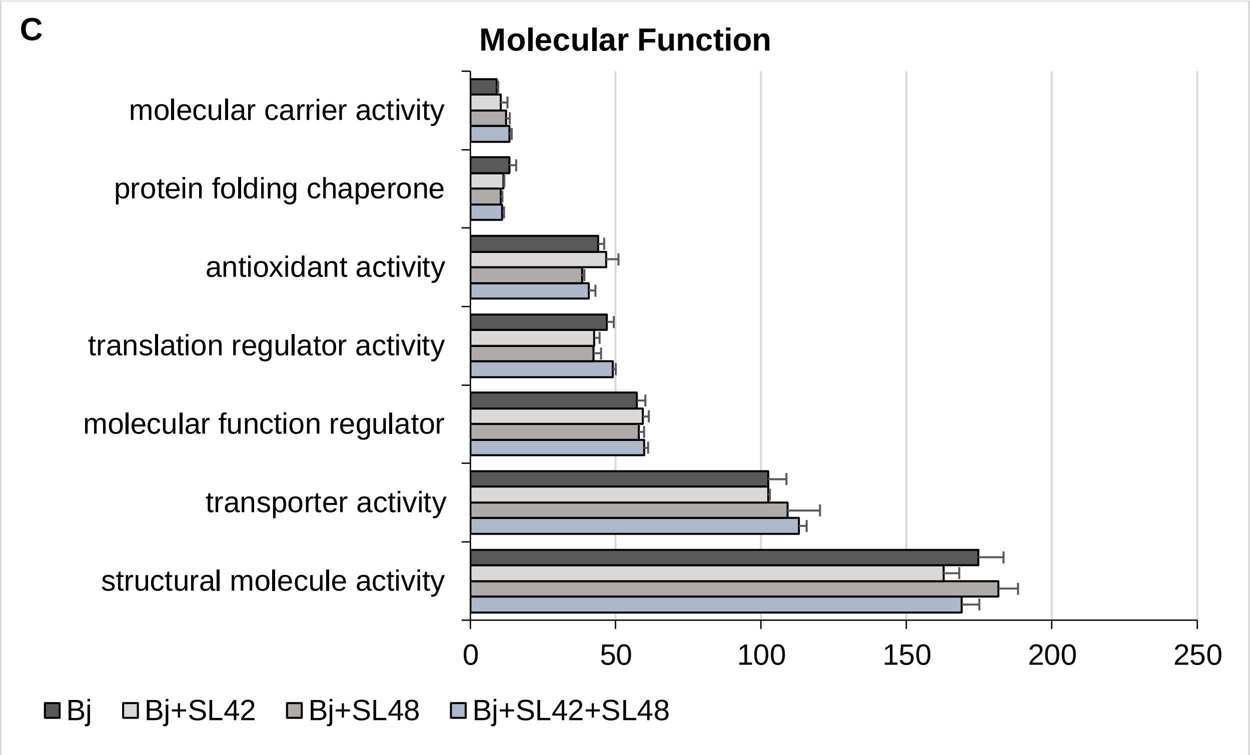

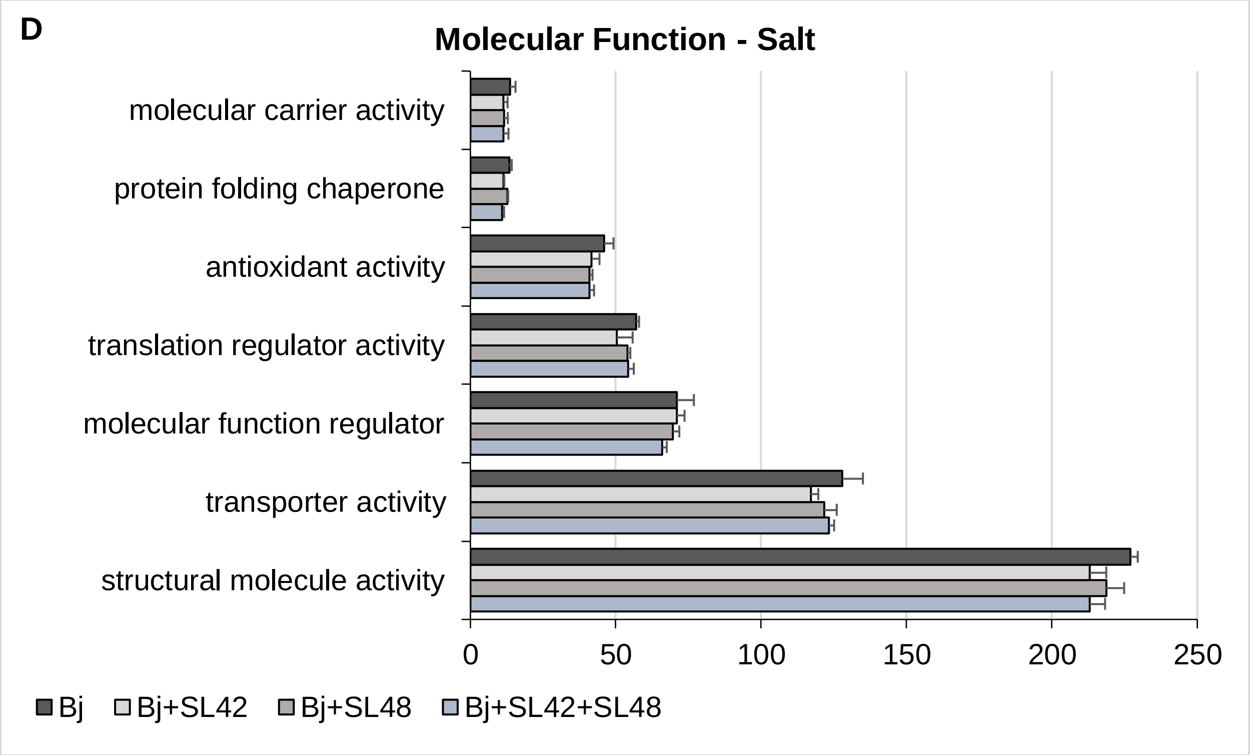

Supplementary Figure S3. (cont.) Number of sequences involved in the molecular functions of the soybean leaf proteome. **(C)** seeds were bacterized with *Bradyrhizobium japonicum* (Bj) as control or the strains *Rhizobium* sp. SL42, *Hydrogenophaga* sp. SL48 were co-inoculated with Bj under optimal and **(D)** under salt stress conditions. Values represent mean ± SE (n=3).

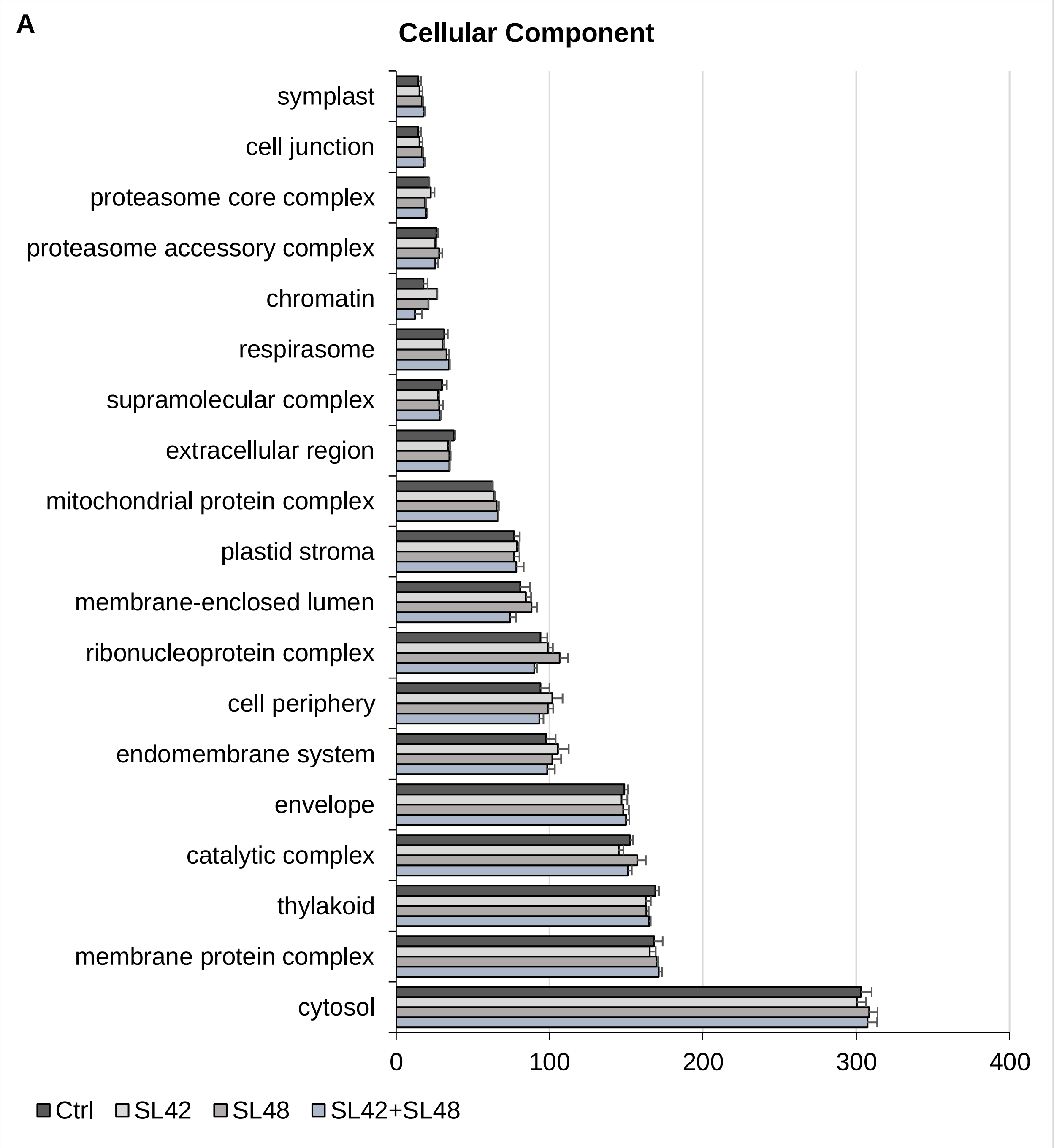

Supplementary Figure S4. Number of sequences involved in the cellular components of the soybean leaf proteome. **(A)** The seeds were treated with 10 mM $MgSO_4$ or bacterized with the strains *Rhizobium* sp. SL42, *Hydrogenophaga* sp. SL48 or co-inoculated under optimal conditions. Values represent mean ± SE (n=3).

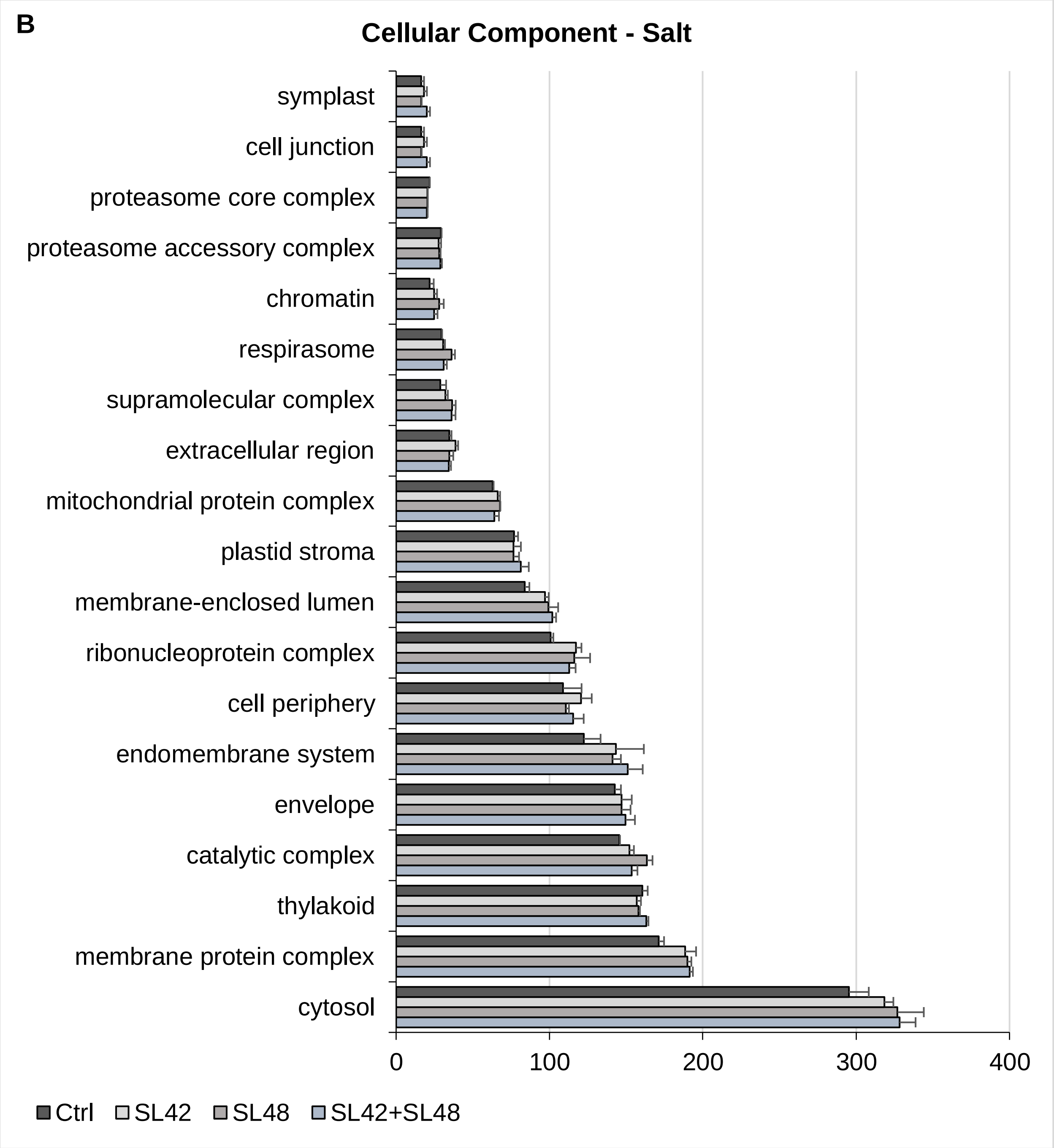

Supplementary Figure S4. (cont.) Number of sequences involved in the cellular components of the soybean leaf proteome. **(B)** The seeds were treated with 10 mM $MgSO_4$ or bacterized with the strains *Rhizobium* sp. SL42, *Hydrogenophaga* sp. SL48 or co-inoculated under salt stress conditions. Values represent mean ± SE (n=3).

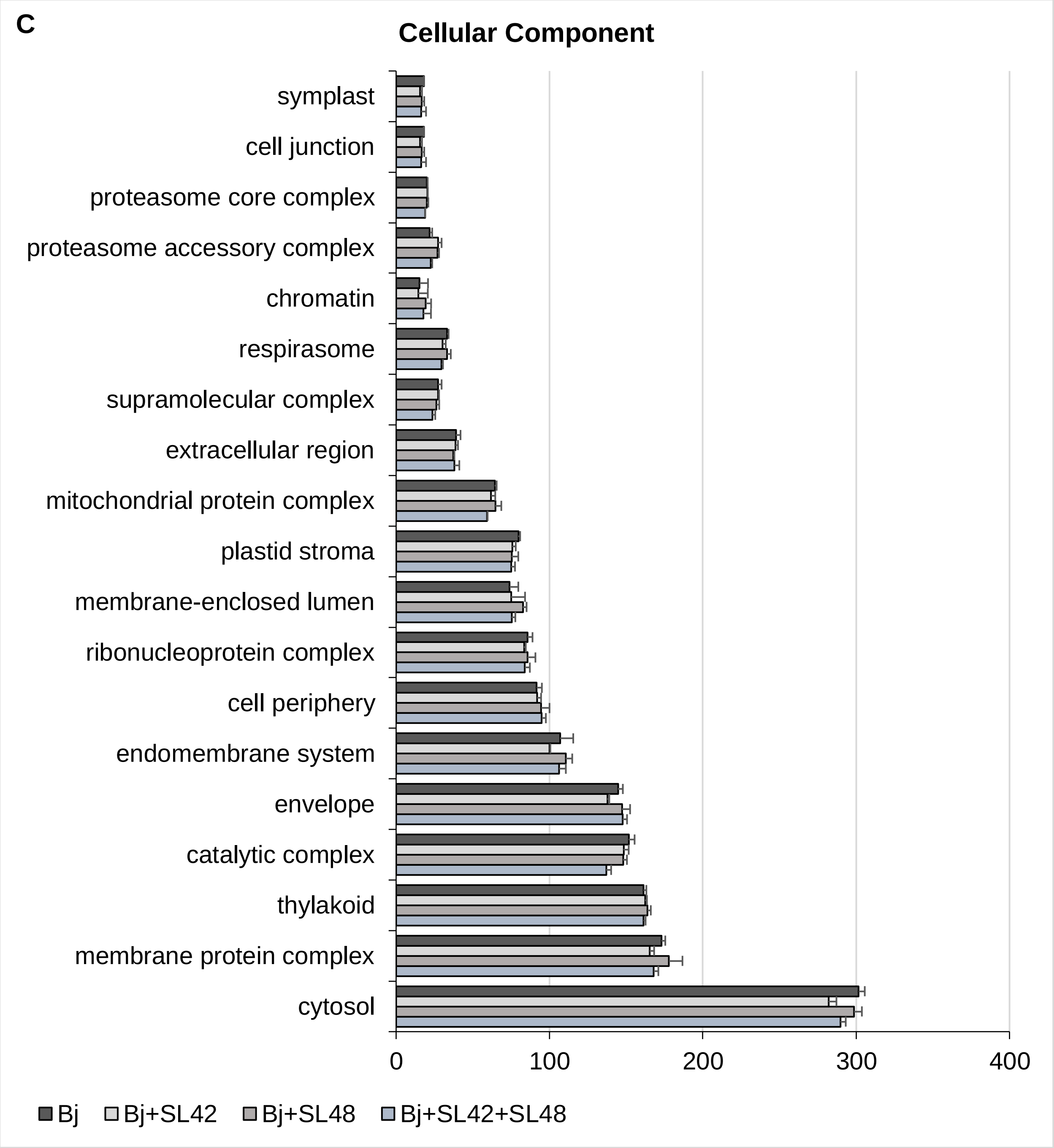

Supplementary Figure S4. (cont.) Number of sequences involved in the cellular components of the soybean leaf proteome. **(C)** seeds were bacterized with *Bradyrhizobium japonicum* (Bj) as control or the strains *Rhizobium* sp. SL42, *Hydrogenophaga* sp. SL48 were co-inoculated with Bj under optimal conditions. Values represent mean ± SE (n=3).

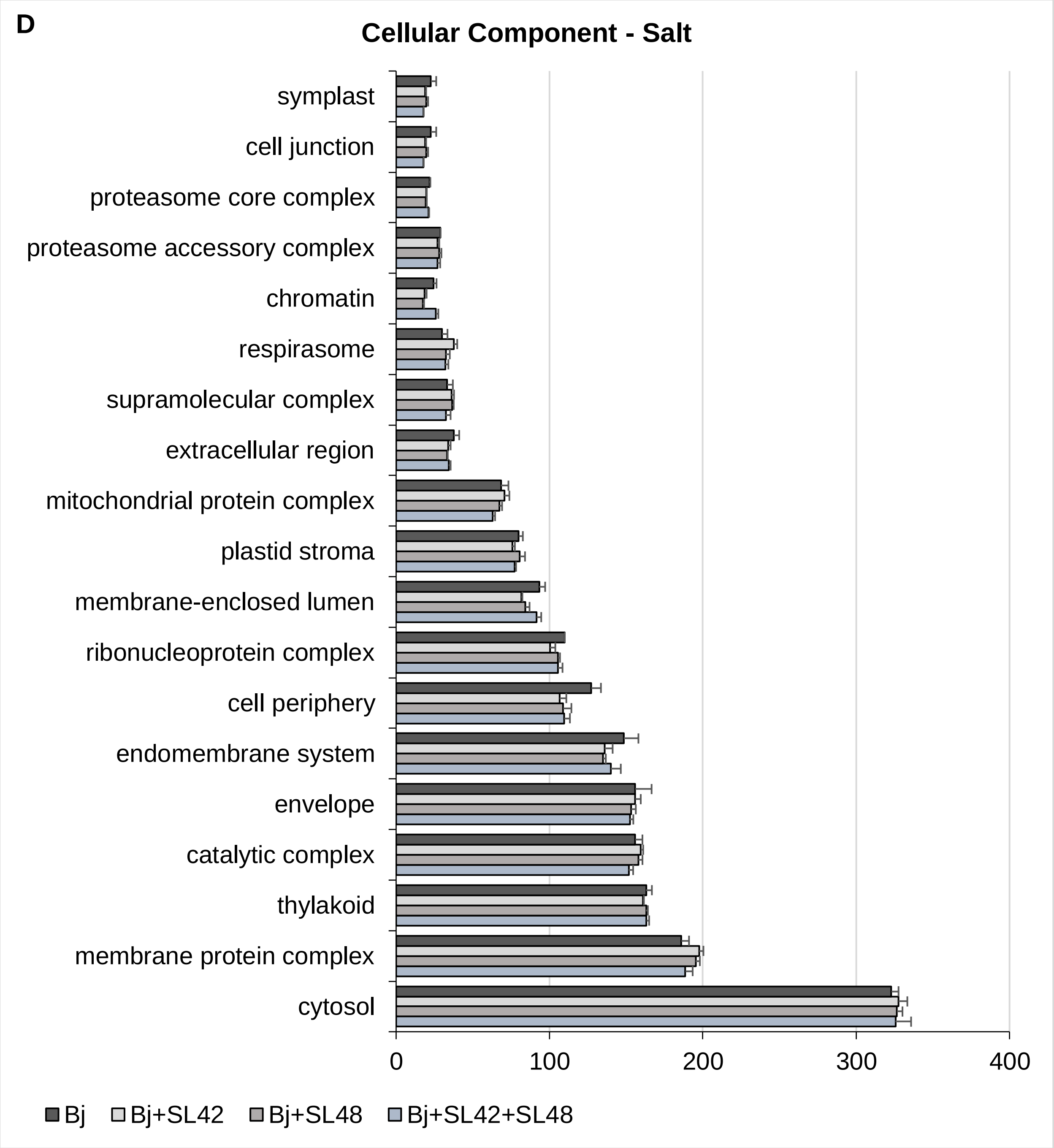

Supplementary Figure S4. (cont.) Number of sequences involved in the cellular components of the soybean leaf proteome. **(D)** seeds were bacterized with *Bradyrhizobium japonicum* (Bj) as control or the strains *Rhizobium* sp. SL42, *Hydrogenophaga* sp. SL48 were co-inoculated with Bj under salt stress conditions. Values represent mean ± SE (n=3).

Functional classification of proteins based on GO categories

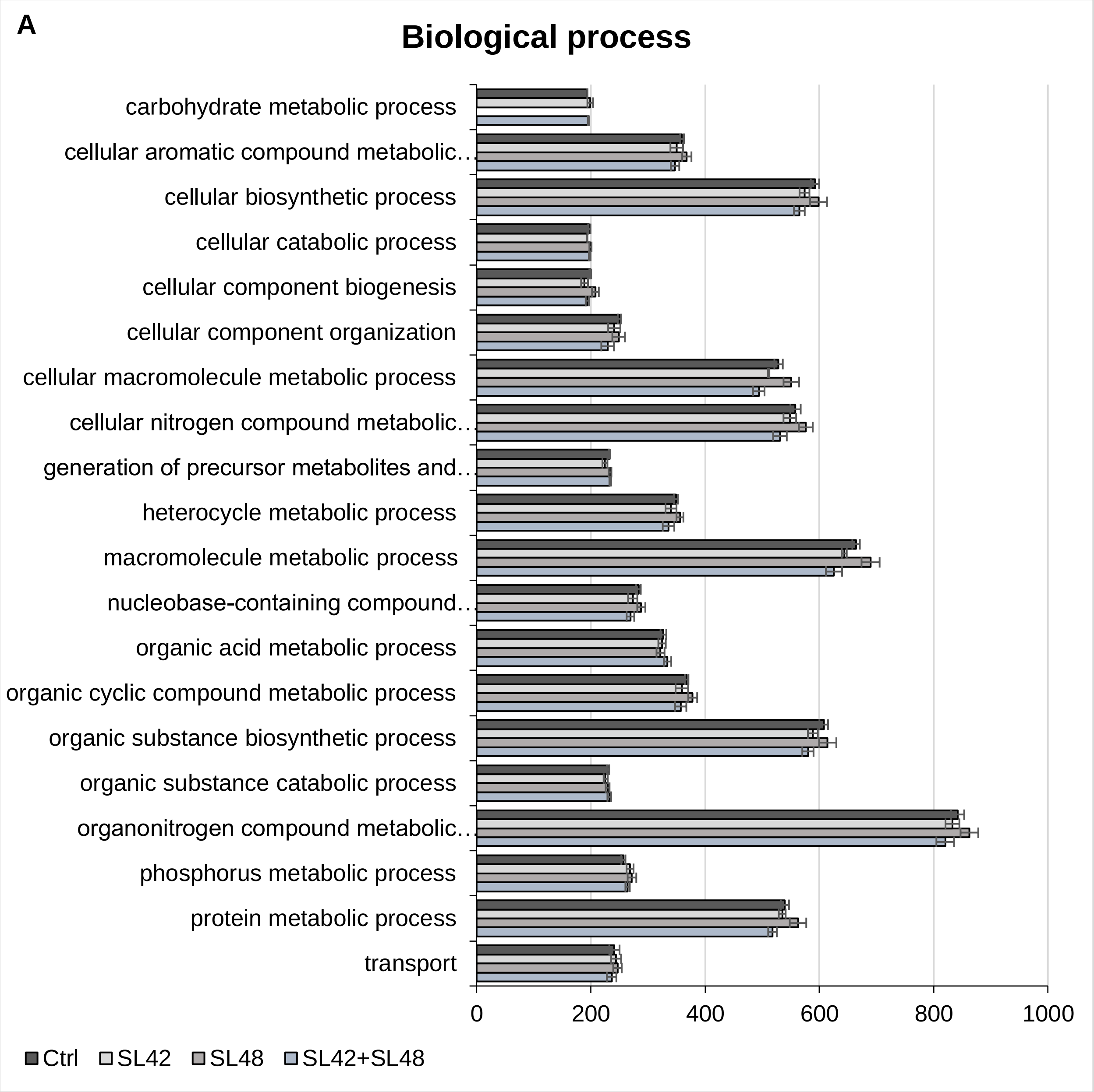

Supplementary Figure S5. Number of sequences involved in the cellular and metabolic processes of the soybean leaf proteome. **(A)** The seeds were treated with 10 mM $MgSO_4$ or bacterized with the strains *Rhizobium* sp. SL42, *Hydrogenophaga* sp. SL48 or co-inoculated under optimal conditions. Values represent mean ± SE (n=3).

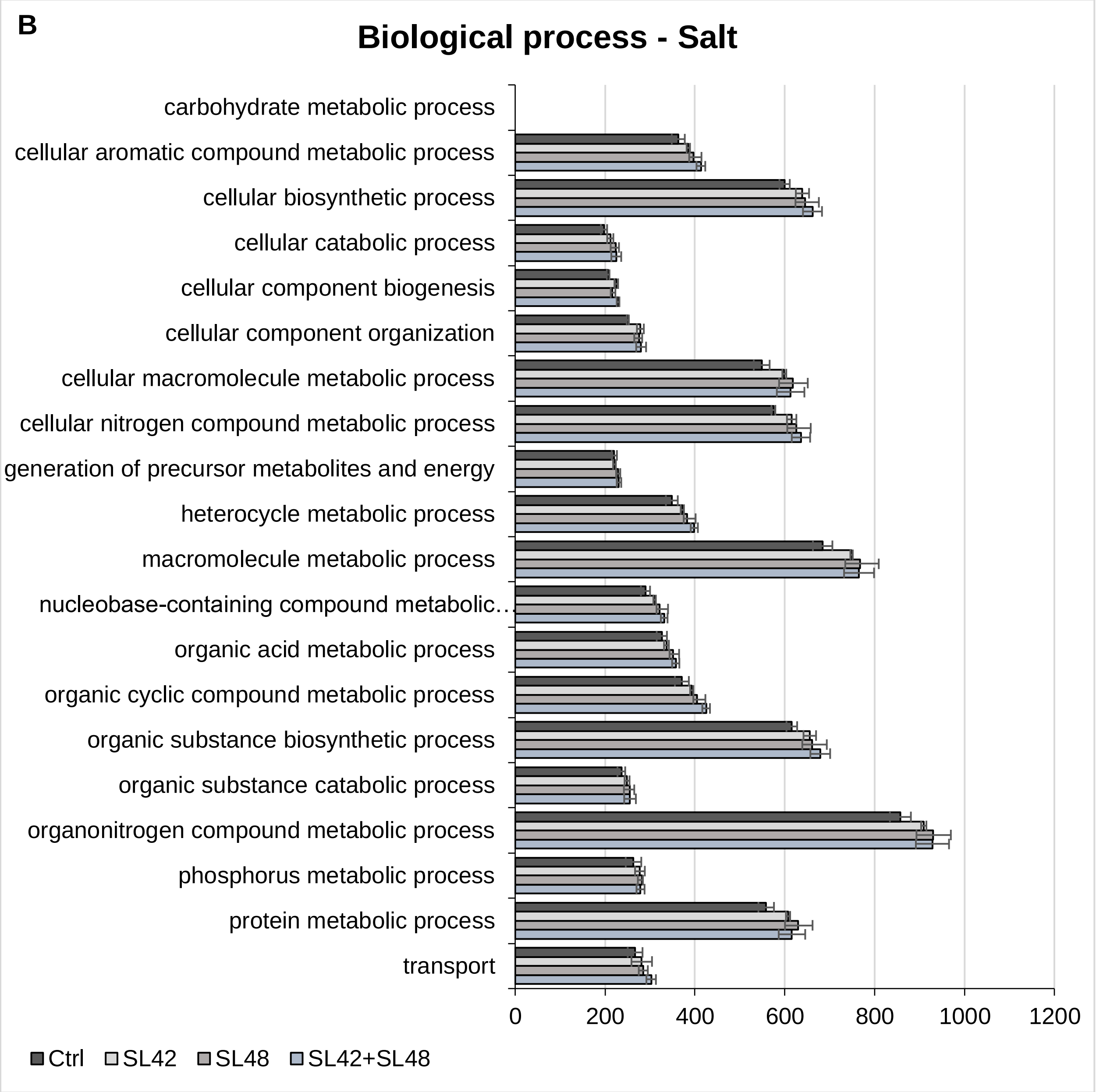

Supplementary Figure S5. (cont.) Number of sequences involved in the cellular and metabolic processes of the soybean leaf proteome. **(B)** The seeds were treated with 10 mM $MgSO_4$ or bacterized with the strains *Rhizobium* sp. SL42, *Hydrogenophaga* sp. SL48 or co-inoculated under salt stress. Values represent mean ± SE (n=3).

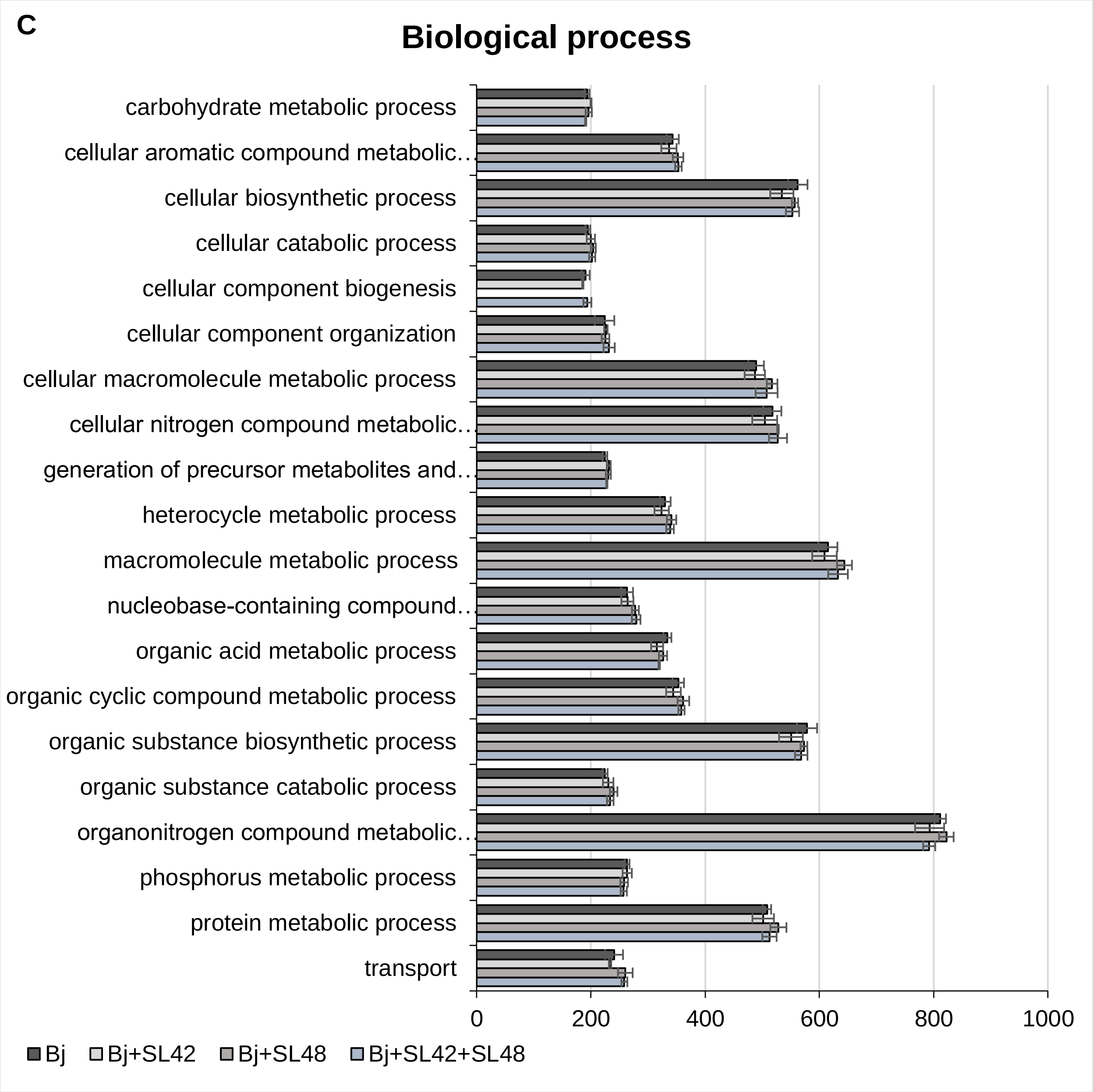

Supplementary Figure S5. (cont.) Number of sequences involved in the cellular and metabolic processes of the soybean leaf proteome. **(C)** The seeds were bacterized with *Bradyrhizobium japonicum* (Bj) as control or the strains *Rhizobium* sp. SL42, *Hydrogenophaga* sp. SL48 were co-inoculated with Bj under optimal conditions. Values represent mean ± SE (n=3).

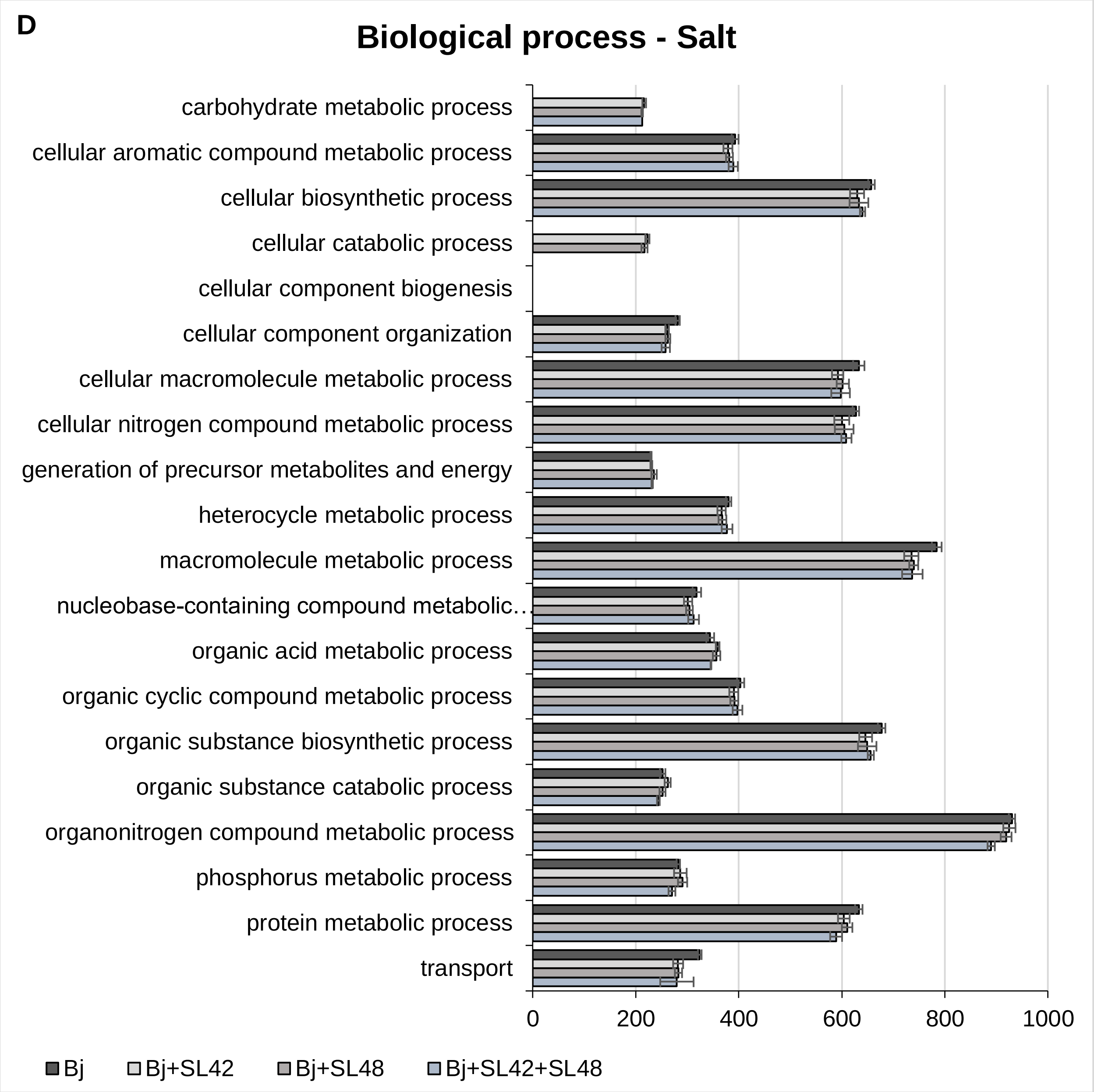

Supplementary Figure S5. (cont.) Number of sequences involved in the cellular and metabolic processes of the soybean leaf proteome. **(D)** The seeds were bacterized with *Bradyrhizobium japonicum* (Bj) as control or the strains *Rhizobium* sp. SL42, *Hydrogenophaga* sp. SL48 were co-inoculated with Bj under salt stress. Values represent mean ± SE (n=3).

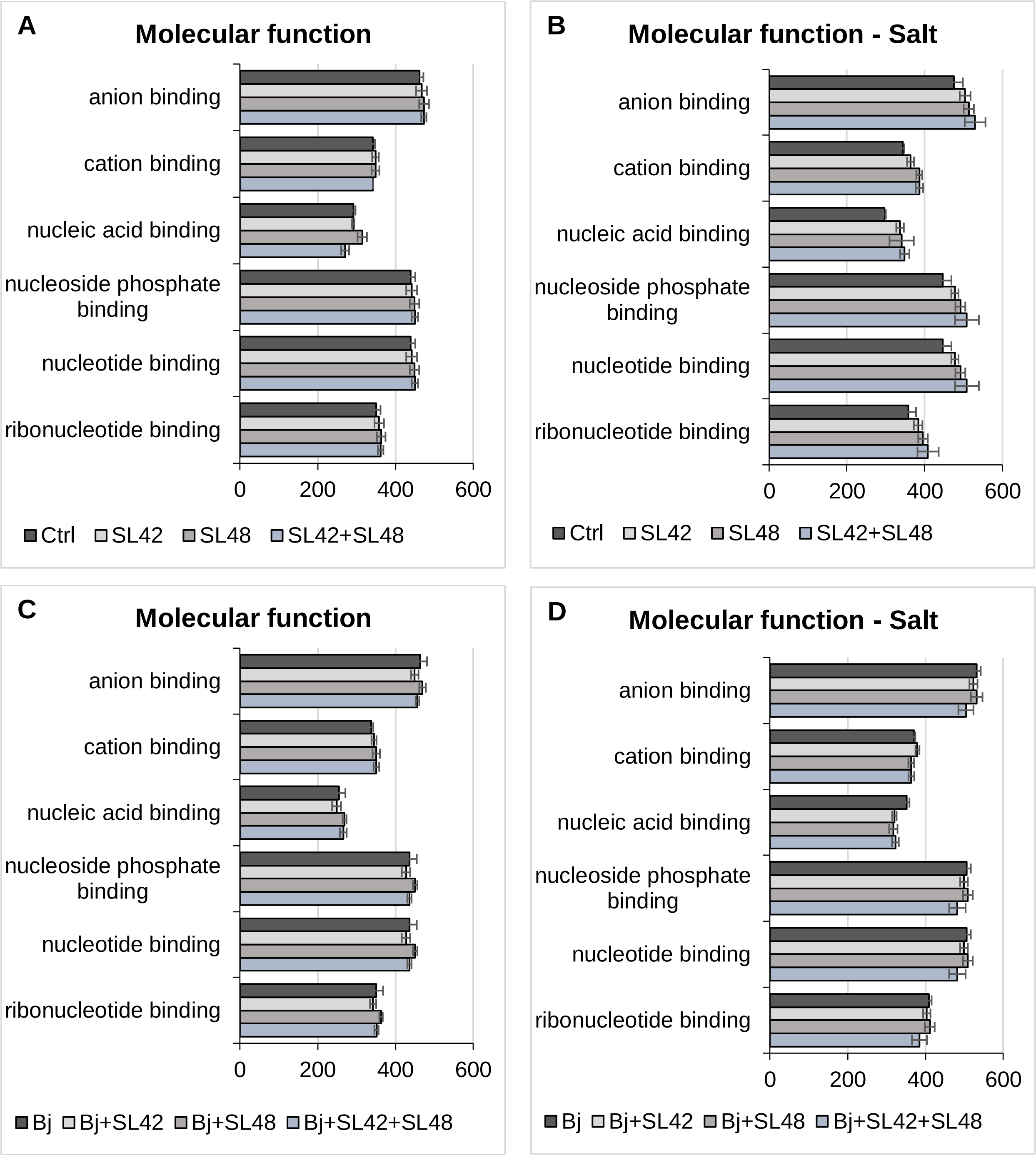

Supplementary Figure S6. Number of sequences involved in the molecular function - binding of the soybean leaf proteome. **(A)** The seeds were treated with 10 mM $MgSO_4$ or bacterized with the strains *Rhizobium* sp. SL42, *Hydrogenophaga* sp. SL48 or co-inoculated under optimal and **(B)** under salt stress or **(C)** the seeds were bacterized with *Bradyrhizobium japonicum* (Bj) as control or the strains were co-inoculated with Bj under optimal and **(D)** under salt stress conditions. Values represent mean ± SE (n=3).

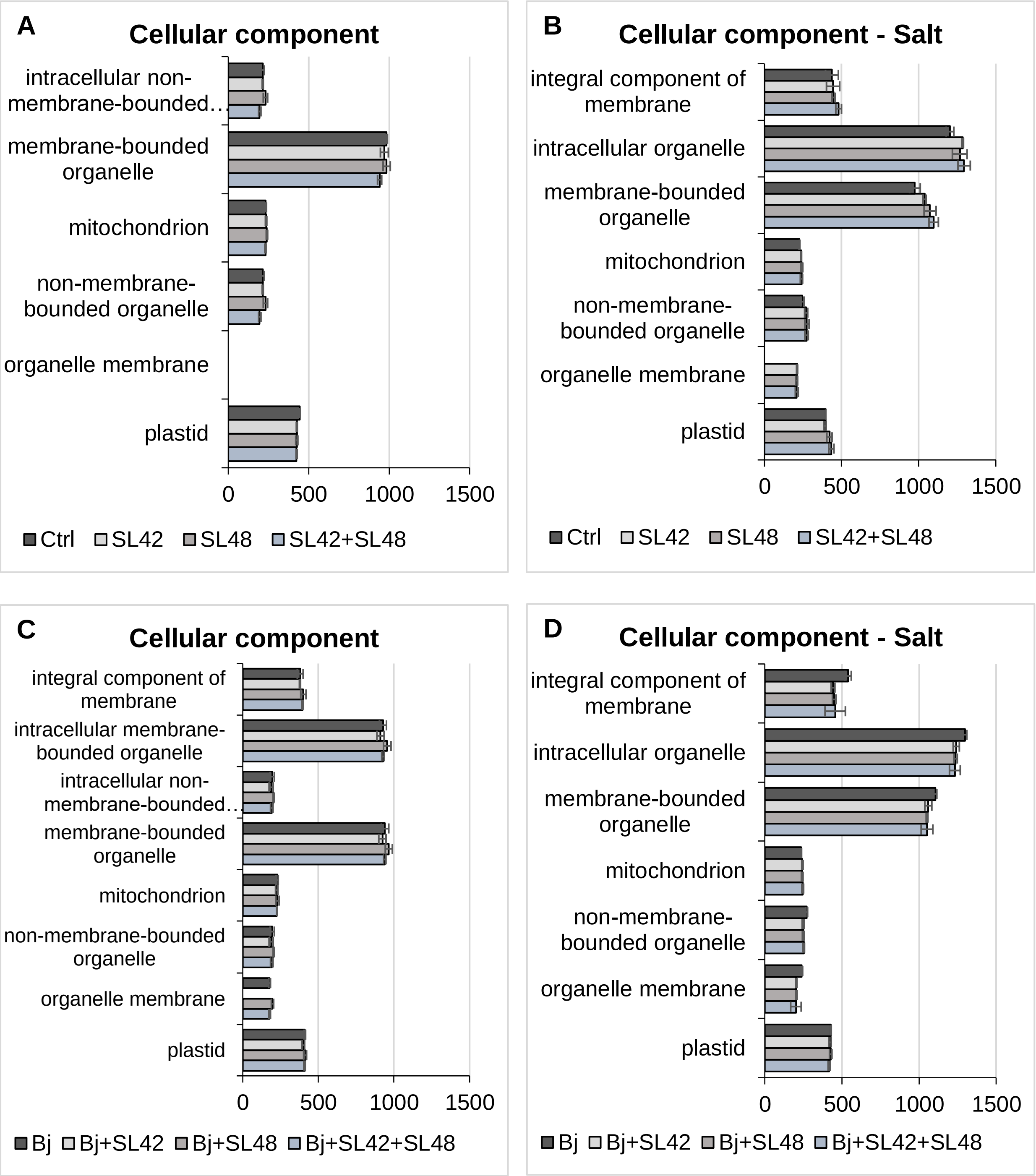

Supplementary Figure S7. Number of sequences involved in the cellular components – membranes and organelles of the soybean leaf proteome. **(A)** The seeds were treated with 10 mM $MgSO_4$ or bacterized with the strains *Rhizobium* sp. SL42, *Hydrogenophaga* sp. SL48 or co-inoculated under optimal and **(B)** under salt stress or **(C)** the seeds were bacterized with *Bradyrhizobium japonicum* (Bj) as control or the strains were co-inoculated with Bj under optimal and **(D)** under salt stress conditions. Values represent mean ± SE (n=3).

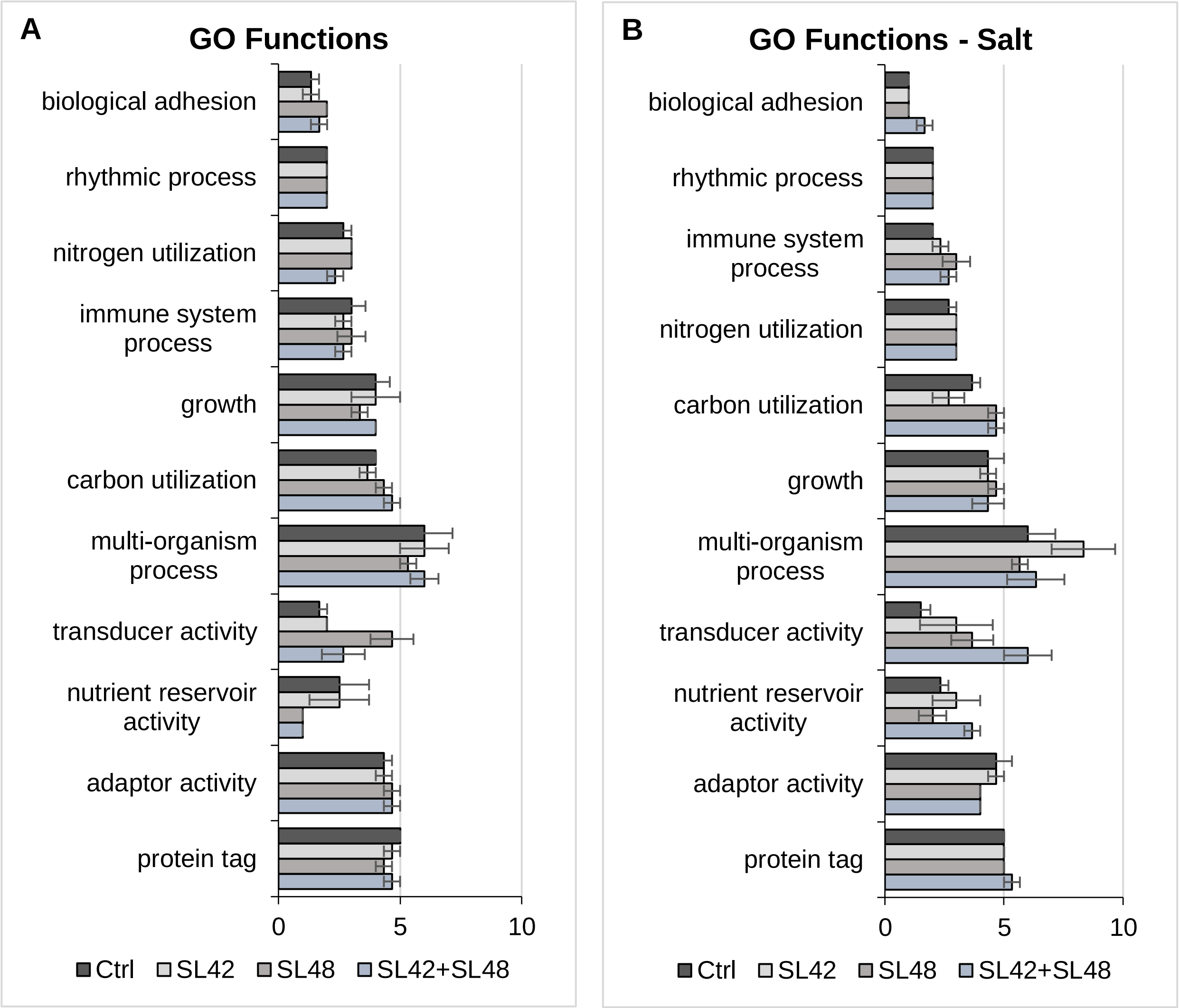

Supplementary Figure S8. Number of sequences (< 10) involved in the GO functions of the soybean leaf proteome. **(A)** The seeds were treated with 10 mM $MgSO_4$ or bacterized with the strains *Rhizobium* sp. SL42, *Hydrogenophaga* sp. SL48 or co-inoculated under optimal and **(B)** under salt stress conditions.

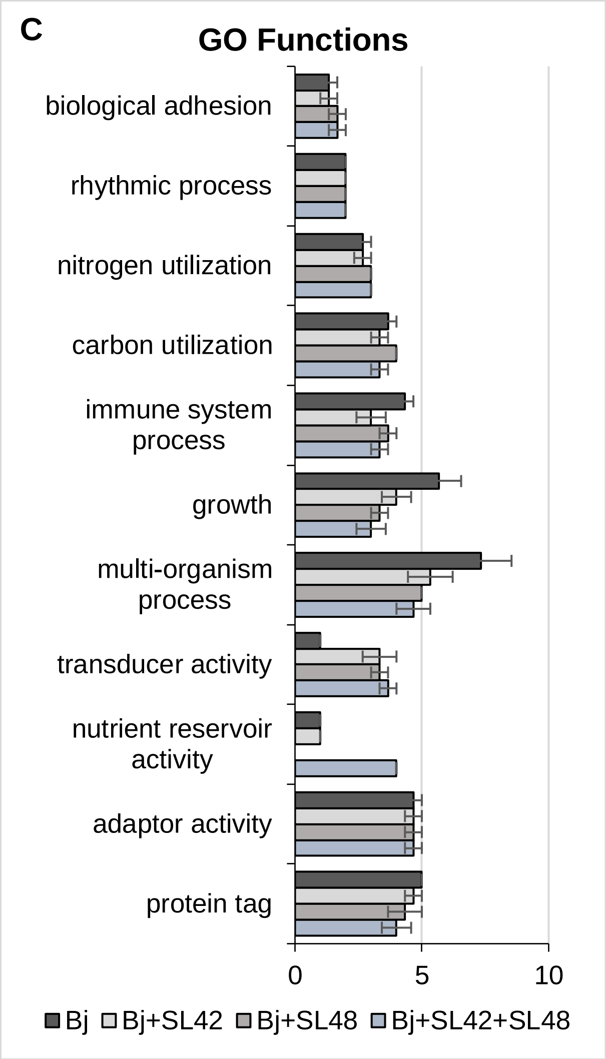

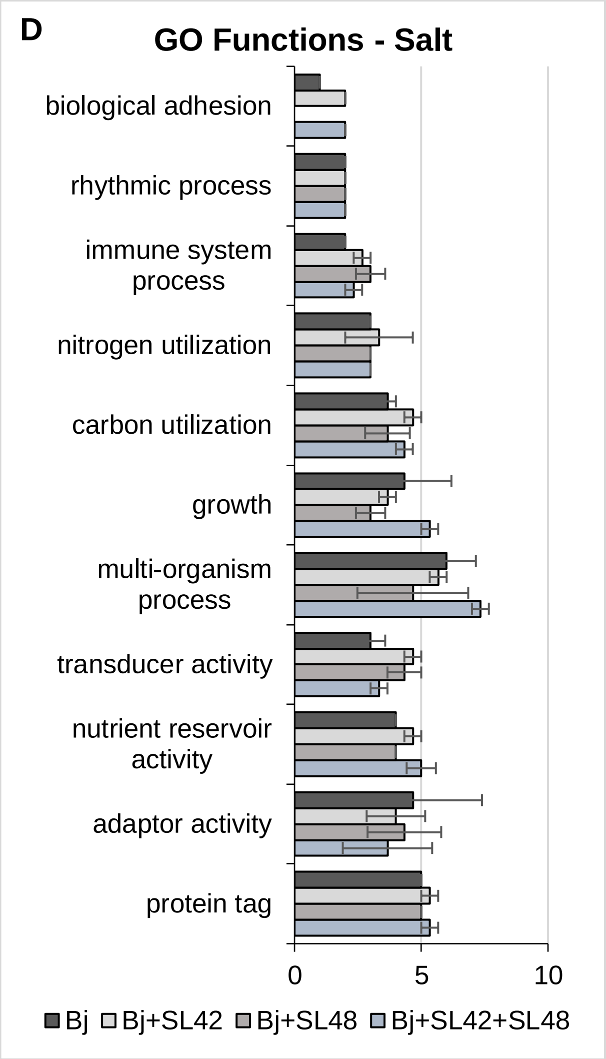

Supplementary Figure S8. (cont.) Number of sequences (< 10) involved in the GO functions of the soybean leaf proteome. **(C)** The seeds were bacterized with *Bradyrhizobium japonicum* (Bj) as control or the strains *Rhizobium* sp. SL42, *Hydrogenophaga* sp. SL48 were co-inoculated with Bj under optimal and **(D)** under salt stress conditions. Values represent mean ± SE (n=3).
